# Unraveling the Adsorptive/Catalytic Roles of Carbonaceous Materials in Per- and Polyfluoroalkyl Substance (PFAS) Degradation: Current Status and Perspectives

**DOI:** 10.1021/acs.est.5c07297

**Published:** 2025-10-01

**Authors:** Justin H. K. Man, Zexiao Zheng, Xiaoying Wang, Howard Y. M. Cheung, Zibo Xu, Jonathan J. Calvillo Solís, Irene M. C. Lo

**Affiliations:** † Department of Civil and Environmental Engineering, 58207 The Hong Kong University of Science and Technology, Hong Kong 999077, China; ‡ Institute for Advanced Study, The Hong Kong University of Science and Technology, Hong Kong 999077, China

**Keywords:** adsorptive/catalytic roles, advanced oxidation/reduction processes, carbonaceous materials, PFAS degradation, thermal treatment

## Abstract

Per- and polyfluoroalkyl substances (PFAS), as persistent environmental pollutants, require advanced degradation technologies beyond conventional adsorption to mitigate their ecological and health risks. With notable adsorptive and catalytic properties, carbonaceous materials have emerged as a potential group of candidates capable of enhancing the PFAS degradation. Hence, a comprehensive understanding of the roles of carbonaceous materials in PFAS degradation is crucial to paving the way for developing efficient and applicable PFAS degradation technologies. This critical review systematically evaluates the physicochemical properties of carbonaceous materials, reveals their roles in different PFAS degradation technologies, and identifies challenges for real-world application. This study reveals that tailored hydrophobicity, surface functionalization, and porosity in carbonaceous materials significantly improve PFAS adsorption, and the rapid charge transfer and generation of charge carriers enable catalytic activity for PFAS degradation. However, limited material stability during application, interference from complex water matrices, toxicity from material leaching, PFAS degradation intermediates, and chemical additives, along with limited system expandability, remain key challenges. By bridging material science with environmental engineering, this review discusses actionable strategies for developing innovative degradation technologies using carbonaceous materials as well as advancing the technologies toward practical applications.

## Graphical abstract

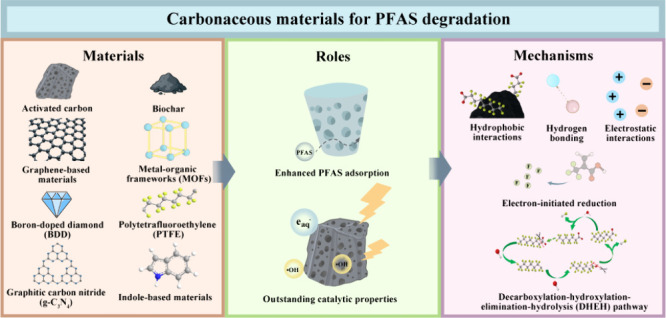

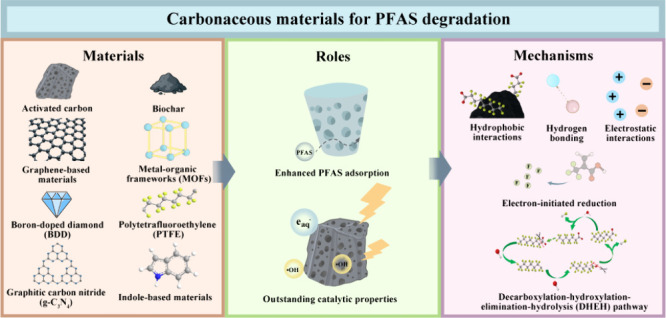

## 1 Introduction

PFAS represent a group of emerging micropollutants which consist of fluorinated compounds containing at least one perfluorocarbon moiety (typically CF_3_– and −CF_2_– groups).^1,2^ Due to the unique stability and surfactant properties, along with their chemical resistance toward corrosion, the applications of PFAS have penetrated every aspect of our daily life, ranging from grease-repelling packaging and waterproof layers to firefighting foams.^3,4^ Common PFAS, including perfluoroalkyl carboxylic acids (PFCAs), perfluoroalkanesulfonates, and hexafluoropropylene oxide dimer acid (HFPO–DA, i.e., GenX), are frequently applied in industries. Owing to the strong C–F bond, the extreme persistence of PFAS implies that they resist degradation in conventional treatment processes and often pass through water and wastewater treatment plants without degradation. Furthermore, their high mobility, notably for short-chain PFAS, limits the effectiveness of the adsorption of PFAS molecules for further degradation. Thus, PFAS have been detected globally in surface water sources.^5–7^ Their persistence in the environment and bioaccumulative nature lead to widespread exposure through contaminated food and water sources,^8,9^ posing health hazards such as immunological disorders, reduced fertility rates, and chronic toxicity.^10,11^ Considering the risks brought about by PFAS pollution, stringent regulations on drinking water standards by governmental agencies have targeted various PFAS. For example, the European Union has regulated the total allowable PFAS level in drinking water as 0.5 μg/L (the list of PFAS included is summarized in Table S1).^12^ Hence, developing innovative strategies for PFAS degradation is essential to safeguarding both the environment and human health.

Physicochemical processes have contributed to PFAS removal. Adsorption, in particular, has been increasingly adopted for the rapid sequestration of PFAS from aqueous environments, which has driven the development and modification of adsorbent materials, such as activated carbon (AC),^13,14^ biochar,^15^ fluoropolymers,^16^ and anion-exchange resins,^17^ to enhance their affinity for PFAS compounds. However, while the adsorbents reach their maximum adsorption capacity, the spent materials and immobilized PFAS still necessitate subsequent treatment, such as incineration, landfill, and regeneration. Owing to the high chemical stability of PFAS, these post-treatment processes not only incur additional operational costs but also pose risks of secondary pollution due to incomplete degradation or the release of toxic byproducts.

In response to the aforementioned limitations, research has shifted toward degradation technologies capable of breaking down PFAS molecules.^18,19^ Advanced oxidation processes (AOPs) (e.g., photocatalysis,^20,21^ electrocatalysis,^22,23^ and piezocatalysis)^24^ advanced reduction processes (ARPs),^25,26^ and thermal treatment^27,28^ have shown potential in PFAS degradation, in which such approaches aim to degrade PFAS to smaller molecules. For instance, photocatalysis, electrocatalysis, piezocatalysis leverage oxidative (e.g., hydroxyl radicals (^•^OH)) reactive species to degrade PFAS, while ARPs leverage reductive (e.g., hydrated electrons (e_aq_^–^)) reactive species for PFAS degradation, progressively fragmenting the carbon backbone and stripping fluorine atoms.^20,29–33^ Additionally, thermal methods apply extreme heat to destabilize C–F bonds adsorbed onto the substrate, degrading PFAS into gaseous or solid residues.^34–36^ It is notable that the ultimate goal for PFAS degradation is to reach complete defluorination (F atom stripping) by C–F bond cleavage and mineralization via C–C bond breaking, forming innocuous end products (e.g., carbon dioxide and fluoride ions). Although a few developed systems have achieved notable mineralization and defluorination performances,^37^ current studies on PFAS degradation have hardly achieved simultaneous defluorination and mineralization completely, leaving room for research on finding suitable materials to facilitate the C–C and C–F bond breaking.

Carbonaceous materials consist of a broad range of substances primarily composed of carbon atoms, such as AC, metal–organic frameworks (MOFs), indole-based material, and biochar. First, carbonaceous materials are used for the PFAS adsorption due to their hydrophobic surfaces, flexible surface functionalities and hierarchical porosity,^38–41^ which potentially contribute to the stabilization of PFAS molecules for rapid catalytic degradation. Such a functionality has motivated interest in leveraging carbonaceous materials for designing concentrate-and-destroy hybrid systems, where adsorption and catalytic processes synergize to amplify PFAS removal efficiency.^42^ On the contrary, as catalytic materials, they also offer abundant active sites and generate charge carriers, enabling robust charge transfer and the production of reactive species for efficient PFAS degradation.^21,43,44^ Further, the chemical and thermal stability of some carbonaceous materials (e.g., biochar) allows long-lasting stability and the possibility of thermal regeneration after long-term operation, suggesting the potential of using carbonaceous material in PFAS degradation.

This Review provides a holistic analysis of the multifunctional roles of carbonaceous materials in advancing PFAS degradation technologies. First, the physicochemical properties of carbonaceous materials are systematically evaluated to establish their relevance in the PFAS degradation processes. The discussion then delves into the adsorptive and catalytic roles with the use of carbonaceous materials across diverse PFAS degradation technologies, aiming to elucidate how their characteristics regulate reaction pathways, including interfacial interactions, electron transfer, and radical generation. The review also identifies key challenges hindering large-scale implementation, such as the long-term stability of materials for real-world applications and the scalability of nanomaterial synthesis. Finally, forward-looking perspectives are proposed to address these gaps. By bridging fundamental insights into technological advancements, this work aims to catalyze innovation in carbonaceous material development for sustainable PFAS degradation.

## 2 Properties and Roles of Carbonaceous Materials in PFAS Degradation

Carbonaceous materials, such as AC, graphene-based materials, MOFs, graphitic carbon nitride (g-C_3_N_4_), boron-doped diamond (BDD), poly­(tetrafluoroethylene) (PTFE), indole-based materials, and biochar, possess various properties that make them potential candidates for PFAS degradation. Figure 1 provides a comparative summary of these materials, highlighting their structural and functional characteristics, as well as their adsorptive and catalytic roles in PFAS removal. The hydrophobicity, tunable surface functional groups and hierarchical porosity of carbonaceous material facilitate PFAS adsorption, promoting the concentration of PFAS in carbonaceous materials. Synergistically, their porosity, enhanced charge transfer ability and the ability to generate charge carriers favor the catalytic degradation of PFAS, making carbonaceous materials indispensable in PFAS degradation.

**Figure 1:**
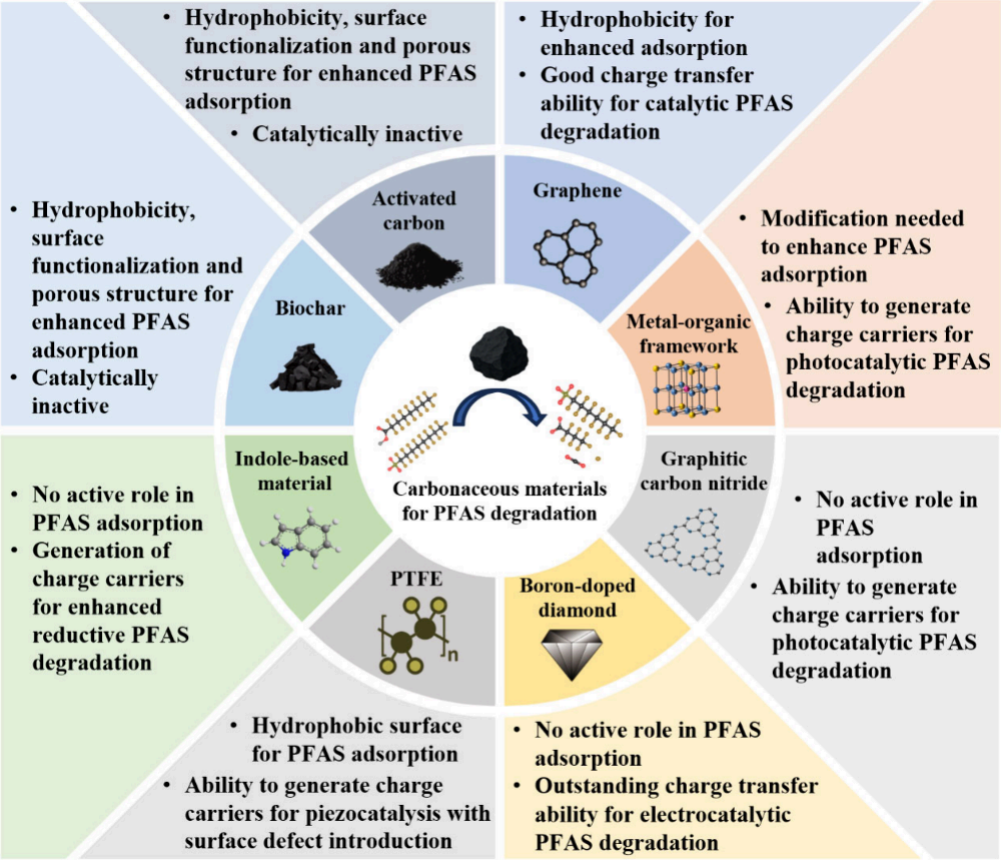
Properties and adsorptive/catalytic roles of carbonaceous materials in PFAS degradation.

### 2.1 Hydrophobicity for Higher Affinity toward Perfluorinated Chains in PFAS

The hydrophobicity of carbonaceous materials, influenced by the chemical structure and degree of surface characteristics within materials, such as AC,^45,46^ graphene-based adsorbents,^47,48^ and biochar,^49–51^ plays a critical role in enhancing PFAS adsorption and promoting pollutant-material mass transfer. Hydrophobic carbon chains enhance PFAS adsorption through mechanisms like van der Waals interactions, hydrophobic effects, and π–π interactions (prominent in AC and graphene), effectively aligning the perfluorinated chain of PFAS with the adsorbent surface.^51^ Since the catalytic degradation of PFAS occurs at the pollutant-material interface, enhanced adsorption of PFAS to the surface of carbonaceous material can subsequently promote mass transfer, thus shortening the transfer distance of reactive species toward PFAS.

Several modification strategies, including chemical engineering, morphological control, and thermal optimization, have been adopted during carbonaceous material synthesis to achieve optimal hydrophobicity. Activated carbon relies on controlled physical/chemical activation methods to preserve its nonpolar surfaces, thereby maximizing van der Waals interactions with PFAS.^52,53^ Graphene-based materials capitalize on their inherent graphitic planes and π–π interactions, with hydrophobicity further refined through reduction processes that minimize oxygen-containing functional groups.^39,48^ Similarly, carbon nanotubes (CNTs) leverage their high specific surface area (SSA) and graphitic walls, synthesized via chemical vapor deposition to ensure structural integrity.^54^ For biochar, the pyrolysis temperature during synthesis is a critical factor, in which higher temperatures (typically 500–700 °C) promote the formation of aromatic carbon structures that enhance hydrophobicity, whereas excessive heating introduces oxidized surface groups that diminish it.^55,56^ These approaches optimize the hydrophobicity of the carbonaceous material, ensuring optimal PFAS preconcentration under operational conditions.^39,52–54^

### 2.2 Surface Functionalization for Higher Affinity toward Ionic Moieties of PFAS

Beyond physical properties, the surface functionalization of carbonaceous materials critically governs their affinity toward the ionic moieties of PFAS. The introduction of functional groups promotes PFAS adsorption via dipole interactions and hydrogen bonds. By the introduction of -NH_2_ and −OH groups to carbonaceous materials (e.g., MOFs and biochar), weak ion-dipole interactions toward anionic PFAS molecules are observed, facilitating the adsorption of anionic PFAS.^57,58^ Further, the oxygen atoms in these functional groups can facilitate the formation of hydrogen bonds with the head of PFAS,^57,59^ affecting the affinity of the carbonaceous materials. Additionally, metal doping introduces metal–oxygen complexes, creating active sites that strengthen biochar-PFAS binding.^60,61^ Optimizing surface functionalization through thermal treatment and metal integration enhances PFAS preconcentration efficiency and degradation potential.

Achieving such surface functionalization on carbonaceous materials relies on strategic modifications and activation pathways, such as physical/chemical activation, pyrolysis tuning, elemental doping, and light activation, which vary between various materials. For AC, which is inherently nonpolar due to its aromatic structure, oxidative treatments during activation (e.g., steam or chemical oxidants) introduce polar functional groups, enhancing surface polarity and creating binding sites for PFAS.^62^ Post-treatment modifications, such as oxidation or nitrogen doping, adjust the surface charge to strengthen electrostatic interactions for anionic PFAS.^63^

### 2.3 Porous Structures for Extra Adsorptive and Catalytic Sites

Porosity, characterized by the presence of interconnected voids spanning micro- (<2 nm), meso- (2–50 nm), and macropores (>50 nm), is a pivotal property of carbonaceous materials that governs their efficacy in PFAS adsorption and catalytic degradation. Hierarchical porosity directly influences SSA, contaminant accessibility, adsorption kinetics, and catalytic site availability.^14,65,67^ Micropores contribute predominantly to high SSA, enabling extensive adsorption via van der Waals interactions and hydrophobic effects as well as increased catalytic sites. Mesopores facilitate the diffusion of PFAS molecules into interior spaces, ensuring efficient utilization of internal surfaces.^64^ Macropores, though less abundant in SSA, serve as transport channels that enhance the mass transfer kinetics, particularly critical for rapid initial uptake.^64,65^ Moreover, the efficacy of carbonaceous materials depends on the relationship between the pore size distribution and PFAS molecular dimensions, and the pores matching the sizes of PFAS molecules can minimize diffusion barriers and amplify confinement effects, promoting both adsorption and catalytic degradation,^66^ as supported by the identification of the mesoporous region of 3–6 nm as the governing mechanism for PFOA adsorption in a study using waste-derived biochar.^67^ Thus, the porosity serves as the structural foundation for both high SSA and functional performance, dictating the balance between adsorption capacity, kinetics, and catalytic activity in PFAS remediation.

Porosity in carbonaceous materials is determined by feedstock selection, synthesis conditions, and post-treatment modifications. Activated carbon relies on physical/chemical activation, by using agents like steam or KOH, to etch hierarchical pore networks and increase SSA.^68,69^ For biochar, pyrolysis temperature is a key condition, in which higher temperatures promote micropore and mesopore formation by volatilizing organic matter,^49,70^ while feedstock selection determines the initial pore architecture.^71^ For instance, biochar derived from woody feedstock has a higher SSA than that of grassy feedstock.^49^ Apart from the conventional biomass sources, recent studies highlight the promise of waste-derived feedstocks, notably sewage sludge and organic waste, which can produce biochar with high surface areas and pore volumes (>1.5 nm) that effectively enhance the affinity toward PFAS, as demonstrated in both laboratory column tests and long-term field lysimeter studies.^72,73^

### 2.4 Charge Transfer Augmentation for Improved Catalytic Performance

Apart from outstanding adsorptive properties, carbonaceous materials, such as graphene and BDD, possess distinct conductive properties, which govern their ability to accelerate charge transfer in catalytic processes. Graphene-based materials exhibit exceptional conductivity due to their unique electronic band structure, characterized by a zero bandgap and a delocalized π-electron network. This enables near-ballistic electron transport with carrier mobilities exceeding 200,000 cm^2^/V·s,^74^ making it a suitable candidate for electrodes and electron mediators in photocatalysts. Unlike graphene, diamond is an insulator with a large bandgap, while boron doping introduces acceptor levels to enhance its conductivity, enabling BDD electrodes to be chemically stable with low background currents and excellent electrochemical properties for PFAS electrocatalysis.^71,72^ The electrochemical properties of BDD enable the generation of reactive oxygen species (ROS) for the chain-shortening and mineralization of PFAS during electrolysis.^76,77^

The conductive properties of carbonaceous materials can be modified by specific strategies. For graphene, intrinsic conductivity arises from its atomic structure, which is enhanced via suitable layer alignment and defect engineering.^78^ Another universal strategy involves hybridizing carbonaceous substrates with nanomaterials to optimize the charge dynamics. For example, coupling graphene with metal oxides or quantum dots (QDs) creates composites that promote charge separation,^79,80^ while BDD composites leverage conductive dopants to enhance interfacial electron transfer.^81^ To further utilize carbonaceous materials for photocatalysis, coupling carbon matrices with semiconductors can enhance the charge transfer ability of the materials, thereby promoting charge carrier separation to increase the formation of reactive species for accelerating PFAS breakdown.^82^

### 2.5 Ability to Generate Charge Carriers for Catalytic PFAS Degradation

Some carbonaceous catalysts can utilize different types of energies to generate electron–hole pairs that drive redox reactions necessary for PFAS degradation. For photocatalysts, which utilize light energy for charge carrier generation, their bandgaps and band positions determine the light absorption spectra and the ability to provide redox potential. For instance, the moderate bandgap of g-C_3_N_4_ (∼2.7 eV) allows visible light absorption, while its conduction and valence band edges (at −1.3 eV and +1.4 eV vs NHE, respectively) enable the generation of reactive species for PFAS degradation.^83^ In addition, the bandgaps of MOFs are highly variable, and can be easily tuned by introducing halogen atoms to the organic linkers.^84,85^ Further, carbonaceous piezocatalysts, notably PTFE, can utilize mechanical energy to drive charge polarization and separation for ROS generation, which is governed by the piezoelectric coefficient that quantifies its efficiency in converting mechanical energy into chemical energy.^32,86^ Furthermore, indole-based materials leverage their aromatic reactivity for functionalization upon ultraviolet (UV) activation, generating e_aq_^–^ for direct defluorination. Oxygen-containing groups like phenolic or carboxylic moieties on indole derivatives promote e_aq_^–^ generation under UV irradiation, cleaving C–F bonds, while quinone moieties act as electron shuttles to boost electron-transfer efficiency.^25,87^

For carbonaceous photocatalysts, modifications aiming to introduce active sites, modulate bandgaps, and improve charge transfer ability have been employed to enhance their photocatalytic reactivity toward PFAS degradation. For example, the introduction of surface defects traps charges, enhancing electron accumulation and surface reactivity.^83^ Conversely, by incorporating π-conjugated linkers and redox-active metal centers, MOFs achieve visible-light absorption and tunable bandgaps; while incorporating halide bridges enhances carrier mobility by enabling electronic coupling between metal nodes.^88,89^ Additionally, the piezocatalytic properties of PTFE are provided by surface defects, as pristine PTFE is centrosymmetric (without piezocatalytic properties).^86^ Hence, by surface engineering or compound fabrication, the piezocatalytic capability of the modified PTFE can be enhanced.^32^

## 3 Carbonaceous Materials in Different Technologies for PFAS Degradation

Recently, carbonaceous materials have been developed for degrading persistent PFAS using several technologies, including AOPs (e.g., photocatalysis, electrocatalysis, and piezocatalysis), ARPs, and thermal treatment. Their intrinsic properties, as detailed in Section 2, critically govern the PFAS degradation efficiency by modulating adsorption and catalytic properties. This section examines how these intrinsic characteristics are strategically leveraged in each technology to enhance PFAS degradation. By correlating carbonaceous material properties with technology-specific performance, the potential limitations in material design and the principles to optimize carbonaceous materials are highlighted, advancing sustainable solutions for PFAS degradation.

### 3.1 Photocatalysis

Photocatalysis has emerged as a potential approach for PFAS degradation, in which carbonaceous materials can either serve as the supporting matrix to other photocatalysts or act as the semiconductor that is photocatalytically active for charge carrier generation. Carbonaceous materials are increasingly recognized for their potential in enhancing the photocatalytic degradation of PFAS (Table S2), overcoming limitations of traditional photocatalysts on wide bandgaps and rapid electron–hole recombination, and poor interaction with PFAS. The integration of carbonaceous materials in photocatalysts could potentially lead to multifunctional advantages, including: (i) their adsorptive capabilities concentrate PFAS closer to photocatalytic active sites, overcoming the poor pollutant-material mass transfer ability;^90,91^ (ii) carbonaceous frameworks broaden the spectral responsiveness to visible-light region with wavelength >400 nm to increase the quantum yield of charge carriers;^91^ (iii) carbon matrices augment charge separation efficiency and prolong the charge carrier lifetime to promote the redox reactions for PFAS degradation;^91–93^ and (iv) increased SSA for enhanced adsorption and increased catalytic sites.^21^

The pathways of photocatalytic PFAS degradation are shown in Figure 2a, which are initiated with the formation and separation of electron–hole pairs in photocatalysts upon light absorption. Electrons further induce the reduction of dissolved oxygen to form superoxide radicals (O_2_^•–^), while holes can oxidize water to produce ^•^OH. These oxidative species (holes, O_2_^•–^, and ^•^OH) can perform photocatalytic degradation of PFAS via the decarboxylation-hydroxylation-elimination-hydrolysis (DHEH) pathway. It begins with decarboxylation by removing the carboxyl group via ROS, followed by hydroxylation, which introduces an −OH group to enhance reactivity. The intermediates then undergo the elimination process by the cleaving of ether bonds or fluorinated fragments, and the hydrolysis process that cleaves the carbon backbone using water, breaking it into smaller molecules.^94,95^ However, upon chain-shortening, the oxidative pathway becomes ineffective in degrading short-chain PFAS. Additionally, the electronegativity of fluorine atoms renders C–F bonds highly resistant to electrophilic attack by oxidative species, limiting their efficacy in defluorination. Thus, while early research on PFAS photocatalytic degradation focused on the oxidative pathway, recent advances have addressed electron-induced reductive H/F exchange, which can directly break C–F bonds.

**Figure 2:**
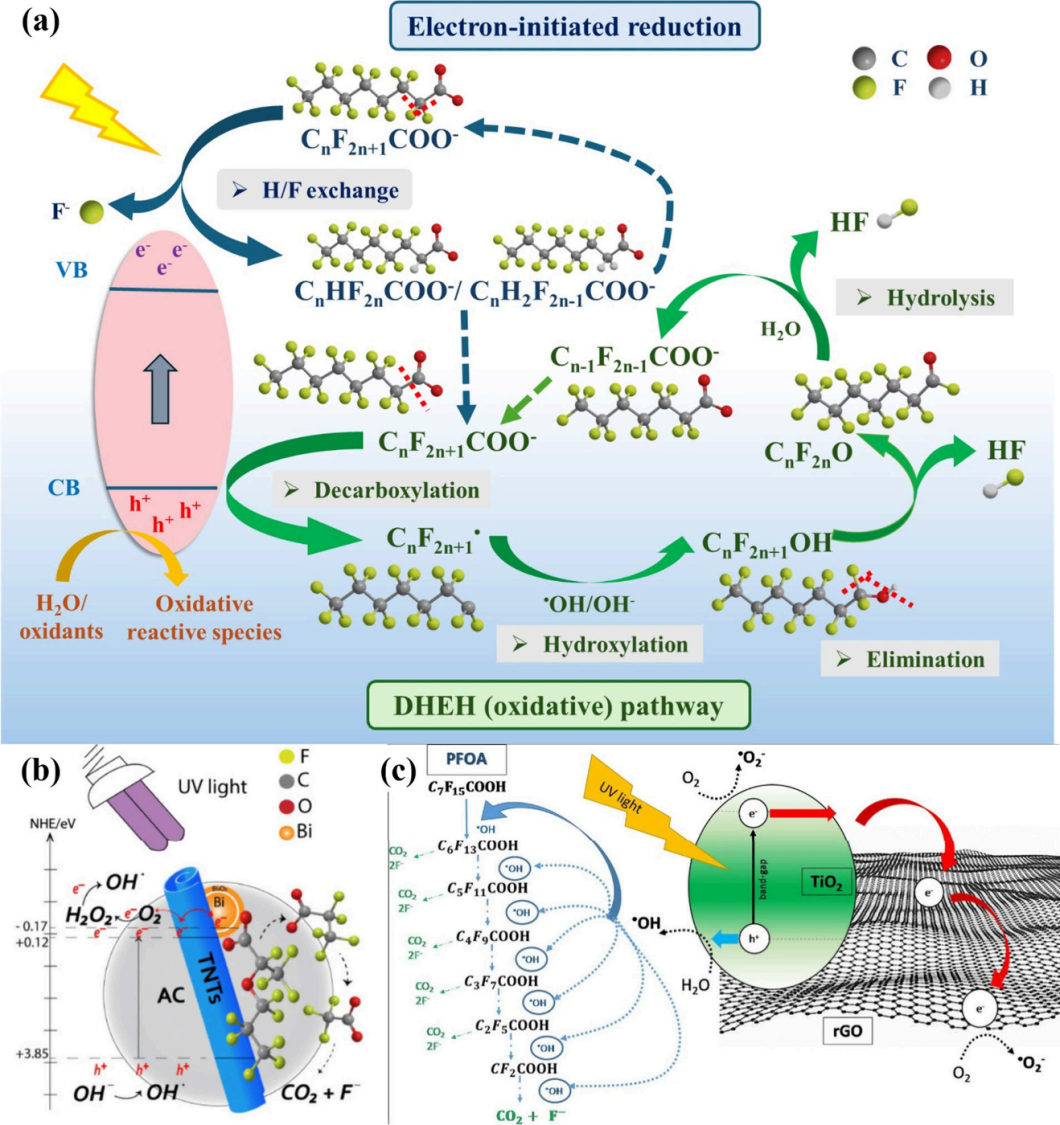
(a) Key pathways of the PFAS degradation via photocatalysis, including the oxidative decarboxylation-hydroxylation-elimination-hydrolysis (DHEH) pathway, and the electron-induced reduction; (b) mechanisms of adsorptive photocatalyst Bi/TNT@AC for GenX degradation; and (c) pathways of the photocatalytic PFOA degradation by TiO_2_-rGO photocatalyst. Figure 2b and c reproduced with permission from refs 96, 97 . Copyright 2022 and 2018 Elsevier Ltd. respectively.

Carbonaceous materials are often employed as supporting matrices to construct composite photocatalysts with semiconductors due to their high PFAS adsorption capacities. Such composites can combine the adsorptive strengths of carbon with photocatalytic capabilities, facilitating efficient PFAS removal through a “concentrate-and-destroy” strategy.^83,85^ In contrast, carbonaceous materials with semiconducting properties are typically employed as photocatalysts due to their ability to generate charge carriers. As carbonaceous materials have been increasingly developed over the years for photocatalytic PFAS degradation, investigating the underlying mechanisms, material innovations, and matrix compatibility of photocatalytic systems utilizing carbonaceous materials is worthwhile.

#### 3.1.1 Photocatalysts with Carbonaceous Materials as Supporting Matrices

Carbonaceous materials, such as AC- and graphene-based materials, CNTs, and hydrophobic polymers, have been developed as effective supporting matrices of semiconductors, such as titanium dioxide (TiO_2_) and indium­(III) oxide (In_2_O_3_), for PFAS degradation, owing to their characteristics of tunable porosity, high hydrophobicity, sufficient functional groups, and outstanding charge transfer ability. Although these materials are not photocatalytically active for charge carrier generation, they enhance the accumulation of PFAS on the surface of hybrid photocatalysts, thus promoting interfacial redox reactions to enhance the overall degradation efficiency. Additionally, their excellent conductivity enables accelerated electron transfer to prevent charge recombination, increasing the availability of electrons and holes for redox reactions. The integration of such a carbonaceous support demonstrates a possible approach to enhance the performance of stand-alone photocatalysts, deserving a critical assessment of their roles in PFAS degradation.

##### 3.1.1.1 Activated Carbon

Recent advancements have integrated AC with titanate nanotubes (TNT@AC), which allows PFAS preconcentration on the photocatalyst surface, enabling synergistic remediation.^94^ TNT@AC, by preconcentrating PFAS molecules onto photocatalytic sites and acting as a high-surface-area scaffold for the anchoring of metal ions, enhances the degradation kinetics by improving mass transfer between the photocatalyst and the PFAS molecules and interfacial charge transfer during degradation.^94,95^ For instance, the use of Bi/TNT@AC photocatalyst enhanced the GenX degradation efficiency to 77.2% in 4 h, compared to the negligible degradation provided using pristine AC and TNT.^95^ The adsorption and photocatalytic mechanisms governing the PFAS degradation are highly intricate. Adsorption occurs primarily through hydrophobic interactions and anion-π interactions between the long hydrophobic chain of PFAS and the AC surface, concentrating pollutants at photoactive sites to enable subsequent degradation.^95,98,99^ Further, metal cocatalysts exhibit enhanced affinity for PFAS carboxylate groups through electrostatic interactions, Lewis acid–base coordination, and metal–ligand complexation, synergistically improving adsorption and catalytic breakdown efficiency.^95,98,100^ Subsequently, the adsorbed PFAS molecules follow the DHEH pathway for the oxidative chain-shortening of PFAS.^94,95^

Metal doping (e.g., Fe, Ga, In, and Bi) in TNT@AC can further enhance the photocatalytic degradation of PFAS by tailoring ROS generation and enhancing charge carrier dynamics to enhance specific reactive pathways. For instance, Fe^2+^ doping promotes the hole-driven decarboxylation of perfluorooctanoic acid (PFOA), directly oxidizing carboxylate groups to initiate chain-shortening via perfluorocarbon cleavage,^94^ leading to partial defluorination and mineralization with shorter-chain PFCAs (C2–C7) formation. Ga/TNT@AC introduces oxygen vacancies that stabilize photogenerated electrons, promoting O_2_^•–^ formation through oxygen reduction, while the holes react with surface-bound H_2_O to yield ^•^OH, which further degrades the perfluorooctanesulfonic acid (PFOS) via hydroxylation and subsequent β-scission of the fluorocarbon backbone.^99,100^ Bi/TNT@AC enhances PFAS degradation via UV-induced surface plasmon resonance effects, which amplify UV light absorption and promote charge carrier generation. Metallic Bi also acts as an electron sink (Figure 2b), transferring electrons to the anatase TiO_2_ phases to suppress electron–hole recombination, thereby extending hole availability for GenX decarboxylation.^95^ The synergy between the adsorption capability endowed by AC and extensive catalytic sites through metal doping ensures the exposure of concentrated PFAS to the generated reactive species, enabling efficient degradation.

##### 3.1.1.2 Graphene

The integration of graphene-based materials into photocatalysis significantly enhances PFAS degradation due to their superior electron mobility, excellent PFAS adsorption capacity, and structural versatility. Meanwhile, graphene functions as an electron mediator under illumination, rapidly transferring photogenerated electrons from semiconductors to its surface, suppressing electron–hole recombination, facilitating electrons to directly cleave C–F bond for defluorination or react with dissolved oxygen for O_2_^•–^ formation, and preserving holes in the valence band of the semiconductors for promoting ROS generation, such as ^•^OH.^97,101^ These ROS induce the degradation of PFAS molecules through DHEH pathways.

Central to this process is the adsorption of PFAS onto the intrinsic hydrophobic surfaces of graphene, facilitated by interactions between the perfluoroalkyl chains and the sp^2^-carbon lattice, as well as π-π stacking with aromatic domains, which is critical for subsequent redox reactions.^74,80^ For instance, sulfonated graphene (SG) in 3D SG-TiO_2_ QD aerogels leverages its hydrophobic edges to adsorb PFOA molecules, reaching 90% PFOA degradation in 10 h, ensuring pollutant proximity to active sites.^80^ Similarly, In_2_O_3_-graphene composites calcined at 400 °C achieve partial graphene detachment, exposing In_2_O_3_ surfaces that maximize PFOA adsorption through bidentate/bridging coordination, achieving ∼95% degradation in 3 h.^102^ In photocatalysts with composite like silicon carbide-graphene quantum dots (SiC-GQD), the hydrophobic SiC and GQD surfaces further enhances PFAS localization near reactive sites, ensuring efficient interfacial contact.^102^ These adsorption-driven processes are foundational to improving degradation efficiency by promoting the interaction of PFAS with catalysts.

The photocatalytic roles of graphene-based materials are underpinned by their capacity to promote interfacial charge transfer, modulate the light absorption ability, and facilitate ROS generation. As an electron sink, graphene suppresses electron–hole recombination by rapidly shuttling photogenerated electrons from semiconductors to its conductive network. For example, 0.5 wt % loading of reduced graphene oxide (rGO) significantly improved the charge separation of the photocatalyst Pb-BiFeO_3_, achieving 48% PFOA degradation in 8 h, markedly higher than that of the unloaded catalyst (no observable degradation).^101^ Besides, GQD has also been reported to form a SiC/GQD composite, which can promote the directional separation of photogenerated electrons to the conduction band of SiC through band alignment, in which electrons can directly reduce PFOS through a H/F exchange pathway, achieving a 93.9% degradation in 20 h.^79^

Moreover, graphene loading extends the light absorption spectrum of photocatalysts into the visible-light region due to its remarkably high light-response. For instance, the use of In_2_O_3_-graphene significantly enhanced light absorption in the visible-light region of 400–800 nm for promoted reactive species formation, resulting in a high PFOA defluorination efficiency of 60.9% in 3 h, higher than that of 29.7% with pristine In_2_O_3_.^102^ Furthermore, the role of graphene in facilitating the quantum yield of reactive species for PFOA degradation was also been reported. In the TiO_2_-rGO composites, rGO functioned as the charge mediator to accept the photogenerated electrons, promoting the accumulation of holes on the valence band of TiO_2_ to improve the production of ^•^OH. Further, rGO also acted as the reactive center of oxygen reduction, where the photogenerated electrons were induced to reduce the accumulated dissolved oxygen on its hydrophobic surface, boosting O_2_^•–^ formation (Figure 2c).^97^

##### 3.1.1.3 Other Carbonaceous Materials

Apart from AC and graphene, other carbonaceous materials, including hydrophobic polymers, carbon spheres, and CNTs also serve as pivotal components in photocatalysts advancing PFAS remediation through synergistic “concentrate-and-destroy” mechanisms. For instance, it has been demonstrated that TiO_2_ composites functionalized with hydrophobic polymers (e.g., polyethylene) achieve efficient PFAS degradation via interfacial adsorption sites, where fluorinated tails stabilize on the hydrophobic surfaces of the polymer and carboxylate heads align with TiO_2_. Such a metal oxide/hydrophobic material interface mitigates chain-length dependency, facilitating the degradation of short-chain PFAS.^92^

Furthermore, Xu et al. engineered an iron (hydr)­oxide/carbon sphere composite (FeO/CS) that adsorbed >99% of PFOA through π-anion coordination and hydrophobic interactions, followed by 57.2% defluorination under solar irradiation.^93^ The incorporation of CS promoted ferrihydrite crystallization, enabling multipoint adsorption and electron transfer from PFOA to Fe­(III).^93^ In parallel, the bismuth phosphate/CS composite (BiOHP/CS) was developed, wherein defect-rich CS facilitated side-on PFOA adsorption, enhancing hole-mediated oxidation and suppressing charge recombination.^90^ The role of the CNT-based photocatalyst was highlighted as well, in which CNTs act as electron reservoirs in TiO_2_ composites, reducing charge recombination losses, extending the light absorption spectrum, and enhancing PFOA adsorption (44% at pH 2) through hydrophobic and hydrogen-bonding interactions.^91^ These findings underscore the versatility of carbonaceous frameworks in tailoring both adsorption and photocatalytic functionalities.

The utilization of carbonaceous materials (e.g., AC and biochar) as the supporting matrices of semiconductors for PFAS degradation has significant limitations. First, photocatalytic activity can be adversely affected by excessive and agglomerated loading of carbonaceous supports in composite materials, particularly when their dosages exceed the optimal value. For example, high graphene loading in Pb-BiFeO_3_/rGO (1.0 wt %) leads to increased light scattering and reduced UV light absorption capabilities to reduce charge carrier formation.^101^ Accordingly, approaches such as layer-by-layer assembly and controlled dispersion are recommended to ensure optimal loading and uniform distribution of carbonaceous materials, thereby balancing the trade-off between enhanced adsorption and conductivity with light scattering.

Second, metal leaching from semiconductors or metal-doping agents can raise toxicological risks to aquatic ecosystems and human health, hindering the applications in real-world scenarios. Tuning the interaction between carbonaceous materials and semiconductors can enhance the material stability to mitigate metal leaching. For example, the negatively charged composite architectures have been shown to reduce metal leaching by exploiting electrostatic interactions between cationic metals and charged surfaces.^85,92^

#### 3.1.2 Photocatalysts with Carbonaceous Materials as Semiconductors

MOFs and g-C_3_N_4_ have emerged as effective semiconductors in hybrid photocatalysts for PFAS degradation. These carbonaceous materials possess visible-light responsiveness and tunable band positions, which can actively engage in photocatalytic reactions by generating charge carriers under light irradiation. Compared to conventional metal-based semiconductors, the characteristics of carbonaceous semiconductors in structural versatility, high SSA with increased active sites, and environmental friendliness with less metal leaching make them more feasible candidates.^89,104^

##### 3.1.2.1 Metal–Organic Frameworks

MOFs have been developed for enhanced PFAS photocatalytic degradation primarily due to their high SSA (1000–10000 m^2^/g), tunable porosity, and intrinsic photocatalytic capabilities associated with tailored electronic structures and active site architectures.^105–107^ The versatility of MOFs, especially their ability to undergo tailored modifications, such as heterojunction construction and functionalization, further enhances their performance in both adsorption and photocatalytic degradation processes.

In terms of their adsorptive roles, MOFs are tailored to optimize the capture of pollutant through increased affinity with PFAS molecules and enhanced mass transfer. For instance, fluorine doping in MOF structures strengthens PFOA adsorption via fluorine–fluorine interactions, highlighting the dominance of chemical affinity as the key adsorptive role.^108^ Similarly, dimensionality reduction, such as converting Ti-based MOFs 3D ZIF-8 into layered 2D ZIF-L, boosts the PFOA adsorption capacity (243.8 vs 177.2 mg/g) and kinetics (1742.9 vs 849.9 mg/g·h) by improving mass transfer and stabilizing adsorbed molecules through interlayer hydrogen bonding.^109^ These structural adjustments maximize the adsorptive sites and interfacial interactions, directly enhancing the adsorption efficiency.

Considering their catalytic roles, MOFs are engineered to enhance photocatalytic capabilities by enhancing charge separation and regulating redox reactions. For instance, coupling an In-based MOF (In-MOF) with bismuth oxyfluoride (BiOF) creates a type II heterojunction that establishes built-in electric fields, where electrons preferentially migrate to BiOF and holes to In-MOF. Consequently, reduced charge recombination and accelerated redox dynamics were achieved, leading to over 95% degradation of PFOA.^110^ Similarly, the introduction of the amino group in MIL-125-NH_2_ (Ti-based MOFs) has been found to play a role in tuning the bandgap to widen the light adsorption region, favoring the generation of e_aq_^–^ for the H/F exchange process, resulting in a desirable defluorination rate of 66.7% in 24 h.^106^

Mechanistic insights demonstrate that the importance of e_aq_^–^ to PFAS degradation.^111^ Nevertheless, single-phase MOFs often suffer from rapid charge recombination, significantly hindering PFAS degradation by e_aq_^–^.^108^ To address this limitation, hole quenchers, like glucose and triethanolamine, have been introduced to deactivate photogenerated holes in MOF-based photocatalysts, thus promoting the separation of electrons.^106,109^ For instance, the addition of glucose to the system can significantly enhance the quantum yield. Additionally, the porous structure of MOFs can influence the hole-quenching efficiency and generate ROS.^110^ Moreover, ROS also contributes to the PFAS chain-shortening processes, which necessitates further mechanistic investigation into the interactions between different reactive species, and to improve the performances.^111^ Through effective hole quenching, a multifaceted approach is used to mitigate rapid electron–hole recombination and to improve photocatalytic performance.

Additionally, coupling peroxymonosulfate (PMS) activation with photocatalytic abilities using transition-metal-based MOFs can regulate the redox reactions to generate highly oxidative sulfate radicals (SO_4_^•–^) for enhanced PFAS degradation. For example, a lignin-based bimetallic (Co/Fe) MOF composite membrane has been shown to generate SO_4_^•–^ upon PMS activation, playing a crucial role in facilitating the decarboxylation process of PFOA degradation under solar light irradiation.^112^ In this system, the transition metal ions (Co/Fe) improve the generation of SO_4_^•–^ and the photogenerated electrons promote the valence cycling of Co/Fe, thus enhancing the overall PFAS degradation efficiency from 20.7% (PMS only) to 89.6% in 3 h.

##### 3.1.2.2 Graphitic Carbon Nitride

As a metal-free semiconductor, g-C_3_N_4_ possesses the unique physicochemical properties of visible-light responsiveness and tunable electronic structure, making it an environmentally benign alternative to conventional UV-dependent photocatalysts.^113,114^ Unlike MOF-based semiconductors, pristine g-C_3_N_4_ suffers from the inherent limitation of low SSA (∼14 m^2^/g), limiting its capacity in PFAS adsorption.^115^ Meanwhile, the shortcomings of g-C_3_N_4_ in rapid charge recombination and low surface reactivity also necessitate strategic modifications to optimize its performance in PFAS remediation.

To increase the SSA and enhance the adsorptive roles of g-C_3_N_4_ toward PFAS degradation, MOFs are often incorporated to construct composite photocatalysts (MOFs-*g*-C_3_N_4_). For instance, the hybridization of g-C_3_N_4_ with MOFs significantly enhanced PFAS capture. Notably, it has been reported that ZIF67 (Co-based MOFs)@C_3_N_4_ composites achieve a SSA of 177.9 m^2^/g, leveraging hydrophobic interactions and electrostatic interactions to adsorb PFOA (47.6% adsorption), promoting its further degradation.^113^ Similarly, coupling g-C_3_N_4_ with MIL-100­(Fe) (Fe-based MOFs) exploits the expansive SSA of MOFs (161.5–177.8 m^2^/g) to concentrate the PFAS molecules via a “concentrate-and-destroy” strategy, which facilitates the transportation of pollutants to photocatalytic active sites.^113,114^ Such a modification also enhances the material’s visible-light absorption within 500–600 nm to boost charge carrier formation for increased ROS yields, promoting chain-shortening reactions.^113^

The catalytic roles of g-C_3_N_4_ have been strengthened using elemental doping and ion addition, which amplify catalytic performance by improving the materials’ charge transfer ability and providing an alternative degradation pathway. For example, fluorine doping introduces nitrogen vacancies and hydrophobic domains in F-doped g-C_3_N_4_, strengthening the interactions with PFAS and ozone during photocatalytic ozonation. This modification increases the PFOA removal efficiency from 57.1% (pristine g-C_3_N_4_) to 74.3%, with ^•^OH and holes identified as dominant oxidants.^114^ Similarly, the iron-doped g-C_3_N_4_ (Fe^3+^/g-C_3_N_4_) photocatalyst leverages visible-light-driven ^•^OH generation to degrade 95% of PFOA within 70 h, achieving near-complete defluorination.^115^ Here, g-C_3_N_4_ stabilizes the Fe^3+^-PFOA complexes, facilitating charge transfer and sequential chain-shortening.

The insufficient stability of the MOF and g-C_3_N_4_ is one of the major concerns when they are applied for PFAS degradation. For instance, MOFs are unstable in acidic wastewater streams as acidic pH levels can induce MOF decomposition through hydrolysis and coordination bond disruption, while the structural stability of g-C_3_N_4_ is negatively affected by photocorrosion and surface oxidation under long-term light exposure.^110,116,117^ To overcome the insufficient stability, MOFs incorporating hard acid metals and hard base ligands have been demonstrated with enhanced stability in mildly acidic environments for PFAS remediation from real wastewater.^106^ As for C_3_N_4_, surface passivation with suitable materials, cocatalyst incorporation, and synthesis conditions optimization to minimize defect density are suggested to improve its resistance against photocorrosion.

In addition, MOFs also face the limitations of low recyclability due to potential structural disintegration under mechanical stress induced by water currents. Recent innovations used lignin-based bimetallic MOF nanofibers to address these issues, maintaining both morphological integrity and catalytic activity over multiple rounds of PFOA degradation under solar irradiation. The lignin/poly­(vinyl alcohol) scaffold endowed the composite with enhanced flexibility and load-bearing capacity, effectively reinforcing the brittle MOF crystals against mechanical degradation.^112^

Incomplete defluorination is another concern, as evidenced by residual shorter-chain PFCAs (e.g., heptafluorobutyric acid) and limited defluorination rate (30–60%), which illustrates the limitation of the DHEH pathway for PFAS degradation.^103^ Some MOFs and g-C_3_N_4_ have highly negative conduction bands suitable for reductive PFAS degradation. Future research should use surface engineering and heterojunctions to enhance charge separation and direct electron transfer for efficient C–F bond cleavage, and further mechanistic studies of these defluorination pathways should also be conducted.

### 3.2 Electrocatalysis

Electrocatalysis has emerged as a probable technology for degrading PFAS, in which carbonaceous materials, either working as anodes or cathodes, have demonstrated unique advantages due to their inherent chemical stability, structural versatility, and tunable surface properties, which collectively enable sustained reactivity under critical electrochemical conditions (Table S3).^118^

As shown in Figure 3a, the electrocatalysis of PFAS by carbonaceous anodes proceeds predominantly through indirect oxidation mechanisms mediated by ROS generated via water electrolysis at the anode surface.^119,120^ The high overpotential of anodes suppresses the oxygen evolution reaction, diverting the charge transfer toward ^•^OH formation. Subsequently, ^•^OH facilitate PFAS chain-shortening via the oxidative DHEH pathway,^23,119^ similar to that of photocatalysis in Section 3.1. In parallel, with the use of carbonaceous cathodes, O_2_ can be reduced to H_2_O_2_, leading to the subsequent Fenton-based ^•^OH generation for indirect PFAS oxidation via the DHEH pathway as well (Figure 3b).^120^ Meanwhile, the direct electron transfer occurring at the cathode can also facilitate the defluorination of PFAS molecules via reduction (Figure 3c).^121^ The specific adsorptive and catalytic roles of the carbonaceous materials in electrocatalytic PFAS degradation are discussed in the following parts.

**Figure 3:**
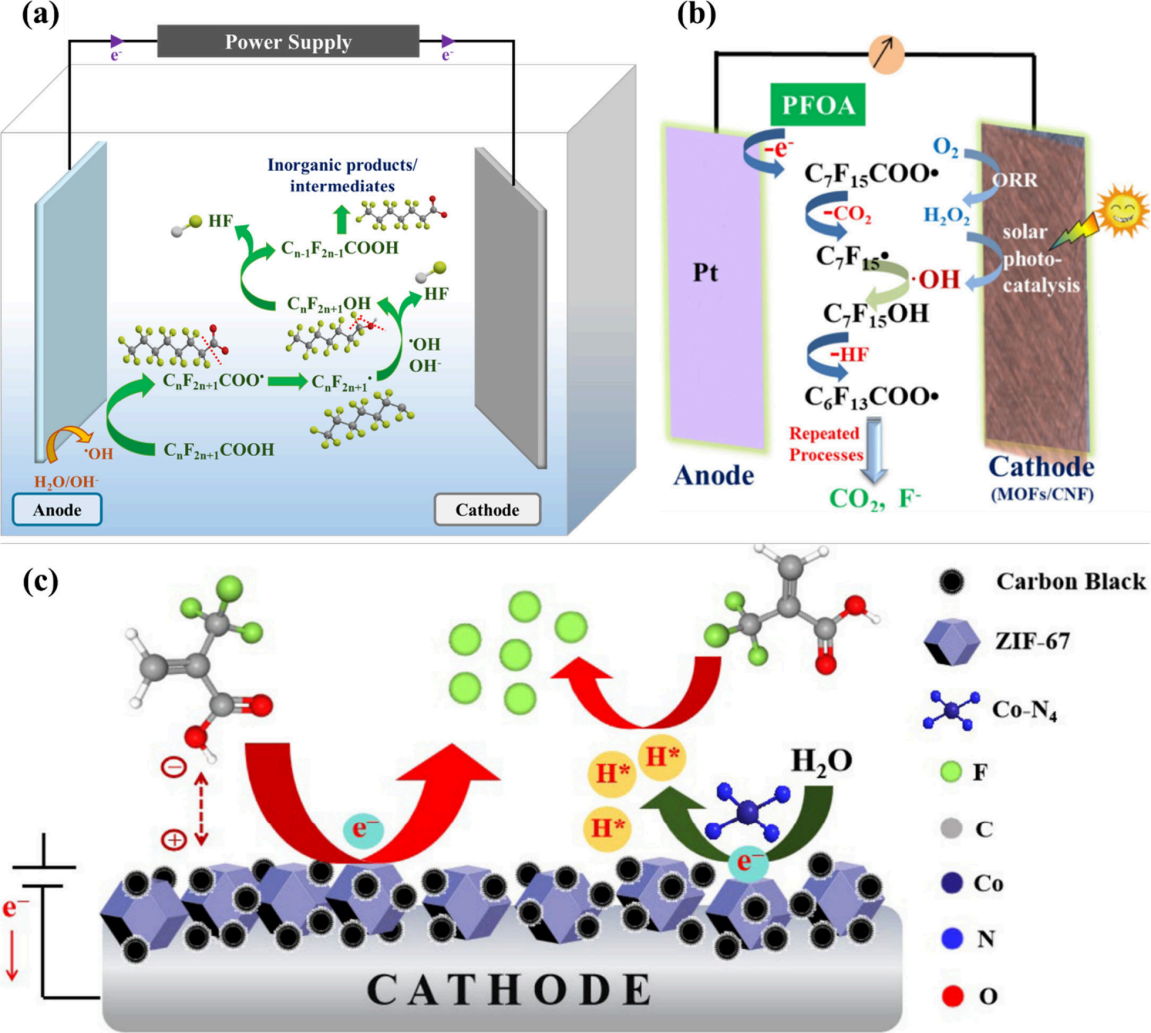
(a) PFAS degradation via oxidative DHEH pathway by electrocatalysis with the use of carbonaceous anode; (b) catalytic role of MOFs/carbon fiber (CNF) cathode in photoelectrocatalytic systems for ROS generation; and (c) direct electrocatalytic reduction of PFAS using MOF-modified cathode. Figure 3b and c reproduced with permission from refs 120, 121 . Copyright 2021 and 2025 Elsevier Ltd. respectively.

#### 3.2.1 Carbonaceous Anodes

Among different carbonaceous materials, BDD-based anodes are suitable for PFAS degradation under extreme conditions due to their high oxygen evolution overpotential, corrosion resistance, and sustained electrocatalytic activity.^122,123^ Unlike conventional metal-based electrodes (e.g., Pt, PbO_2_), BDD demonstrates superior durability, operating effectively at extreme anodic potentials without structural disintegration.^122,123^ The chemical inertness of the diamond lattice resists corrosion even in acidic or high-salinity environments, ensuring consistent performance in different real-world scenarios.^81,124^ The incorporation of boron into the diamond lattice induces p-type conductivity by creating acceptor states, significantly enhancing electron transfer kinetics and enabling sustained high current densities essential for ROS generation.^119,122^ The optimal boron doping levels have been shown in the range of 10^3^ to 10^4^ ppm, which can balance electrocatalytic efficiency and mechanical stability, maximizing conductivity while preserving structural integrity.^119,123^ This structural modification preserves the inherent chemical inertness of the sp^3^-hybridized carbon framework while mitigating electrode fouling, a critical advantage in long-term applications.^22,81^

Differing from other carbonaceous materials, BDD itself cannot adsorb PFAS due to its nonactive nature. As a result, BDD exclusively provides catalytic roles in electrocatalytic PFAS degradation, and does not involve direct redox reactions on the material surface, thereby reducing surface passivation and maintaining catalytic activity over extended periods.^81,125^ Comparative studies demonstrate that BDD achieves near-complete mineralization, surpassing graphene-based alternatives that exhibit lower oxidative potential or faster fouling.^126,127^ Hybrid systems integrating BDD anodes with graphene- or CNTs-based cathodes exploit synergistic effects, combining the high overpotential of BDD with the superior SSA of graphene to enhance the ROS yield and reduce energy consumption.^127,128^ For instance, the graphene-coated nickel foam cathode paired with BDD anodes demonstrates enhanced mass transfer and current density. Considering GenX degradation, such a setup achieved 92.2% of mineralization within 6 h at 16 mA cm^–2^, far exceeding Pt-based anodes (<10%), while reducing energy consumption 10-fold compared to using BDD alone (37.5 vs 237 kWh m^–3^). This synergy arises from BDD-driven electron transfer initiating the cleavage of O-R bond in GenX, followed by Fenton-derived ^•^OH mineralizing intermediates.^127^ Enhanced mass transfer from the 3D structure of graphene further boosts efficiency, positioning hybrid systems as feasible solutions for PFAS degradation.^125,126^

Despite their ability in PFAS degradation, BDD anodes face the limitations of sustained high current densities required for ROS generation, leading to high energy consumption and risks of parasitic side reactions.^120,122^ Future efforts should prioritize innovations such as pulsed electrocatalytic systems that could modulate voltage inputs to optimize ROS generation while minimizing energy losses and electrode wear.^125,128^ Additionally, industrial-scale deployment demands uniform electrode materials with precise boron distribution and nanostructured surfaces to ensure a consistent catalytic performance. Advances in chemical vapor deposition techniques enable scalable synthesis of tailored materials, which is recommended, offering control over the morphology and composition,^124,129^ thereby facilitating cost-effective manufacturing.

#### 3.2.2 Carbonaceous Cathodes

Carbonaceous materials, such as graphene-based structures, hierarchically porous carbons, carbon nanofibers (CNF), and AC, have been developed as cathodes for PFAS degradation, demonstrating versatile roles in electrocatalytic PFAS remediation, primarily through adsorptive and catalytic mechanisms. These carbonaceous cathodes play pivotal adsorptive roles in PFAS degradation, leveraging their intrinsically high SSA and chemically tunable surface functionalities to concentrate on contaminants at reactive interfaces.^22,130^ Such an adsorptive capability is governed by van der Waals interactions and electrostatic interactions, which facilitate the accumulation of PFAS onto the material matrix. A representative example is the hierarchically porous carbon cathode synthesized from MOFs with a multimodal pore architecture: micropores maximize adsorption capacity by providing abundant binding sites; mesopores enable efficient electrolyte infiltration and ion transport; and macropores mitigate mass transfer limitations.^130^ These porous structures synergistically concentrate PFAS near the electroactive regions to prime subsequent degradation pathways. Similarly, AC cathodes exploit their conductive porous networks to immobilize PFAS, effectively reducing diffusion distances to catalytic sites and enhancing degradation kinetics.^22,129^

Carbonaceous cathodes also play a robust catalytic role in the electrocatalytic degradation of PFAS by structural engineering and electronic modifications. It is illustrated that strategic heteroatom doping introduces defects and catalytically active sites, which promote the electrocatalytic degradation of PFAS. As exemplified by the development of nitrogen-doped graphene as reactive electrochemical membranes (REMs), the nitrogen doping not only improves interfacial charge transfer but also promotes the generation of ROS, which mediates the PFAS chain-shortening reactions during electro-Fenton processes.^130^ Furthermore, by introduction of MOFs on carbonaceous cathodes, the reductive defluorination of PFAS via direct electron transfer is facilitated. For instance, compared to pristine carbon paper (CP) cathode, the use of ZIF67 (Co-based MOF) modified CP cathode improved the electrocatalytic defluorination rate of 2-(trifluoromethyl) acrylic acid (4-carbon PFAS) from 9.43% to 97.16% in 48 h,^121^ potentially reducing PFAS intermediate formation. As current studies focused primarily on the defluorination of branched short-chain PFAS, further study of the electrocatalytic reduction of linear long-chain PFAS is necessary.

Moreover, surface functionalization and heterojunction construction also lead to promoted charge transfer for enhanced catalytic degradation. For instance, the functionalization of the CNF with Fe/Co bimetallic MOFs serves as conductive scaffolds and photoactive platforms. As illustrated in Figure 3b, under irradiation, MOFs generate electron–hole pairs, with the CNF matrix directing electrons toward cathodic H_2_O_2_ synthesis and subsequent ^•^OH generation, while photogenerated holes oxidize adsorbed intermediates.^120^ Complementary strategies, such as N-GS/CeO_2_@Ti_4_O_7_ heterojunctions, enhance interfacial charge transfer efficiency and operational stability, suppressing recombination losses and maximizing catalytic site accessibility.^128^ These catalytic mechanisms, spanning electrochemical, photochemical, and hybrid modalities, highlight the versatility of carbonaceous materials in mineralizing PFAS

The interplay between adsorption and catalysis is central to advanced remediation platforms. REMs, with the use of N-GS/CeO_2_@Ti_4_O_7_ synergize the adsorption capacity of nitrogen-doped graphene with continuous electrocatalysis under hydrodynamic flow, while heterostructures improve interfacial charge transfer.^128^ In another case, AC cathodes, apart from adsorption, exploit their conductive frameworks to sustain defluorination via direct electron transfer.^129^ Such systems underscore the transformative potential of carbonaceous materials in facilitating the adsorption.

Generally, carbonaceous cathodes confront difficulties such as rapid electrode fouling by adsorbed organic intermediates (e.g., short-chain PFAS), which block active sites and reduce efficiency.^22,131^ Specifically, system designs utilizing adsorption-catalytic PFAS degradation like REMs require precise balancing of adsorption capacity and catalytic activity, complicating process optimization.^128^ Future advancements should focus on material innovations such as antifouling coatings and defect-engineered carbons to enhance stability and selectivity. Flow-through reactor designs are also recommended, which can mitigate mass transfer limitations and achieve rapid PFAS removal (<10 min hydraulic retention).^128^

### 3.3 Piezocatalysis

Piezocatalysis exploits mechanical stress to induce charge separation in piezoelectric materials (piezocatalysts) upon mechanical stress (e.g., ultrasonic vibration), in which recent advancements have emphasized the role of carbonaceous piezocatalysts, notably PTFE, in the piezocatalytic process for enhanced PFAS degradation. Under mechanical activation, piezocatalysts are polarized with an intrinsically generated electric field, creating electron–hole pairs that drive reduction reaction and ROS generation (Figure 4), following the above-mentioned DHEH pathway for PFAS chain-shortening.^132,133^ With surface defects introduced, PTFE is a novel piezocatalyst due to its exceptional piezoelectric coefficient (d_33_ ≈ 600 pC/N) and structural resilience.^32,134^ Its chemical inertness and thermal stability ensure durability under extreme operational conditions. For its adsorptive role, the hydrophobic surface facilitates selective adsorption of PFAS via fluorophilic interactions, making it uniquely suited for PFAS remediation.^32^ Considering the catalytic role, under mechanical stress, the piezoelectric response of PTFE generates ROS, which degrade the adsorbed PFAS through sequential defluorination and mineralization.^86,134^ For instance, the use of surface-defect-induced PTFE piezocatalyst has achieved complete degradation and high defluorination rate (91.5%) for PFOA by aligning PFAS chains with catalytic sites to optimize electron transfer.^32^ The combination of adsorption-driven targeting and piezocatalytic activity positions PTFE as a potential solution for chemically free PFAS degradation.

**Figure 4:**
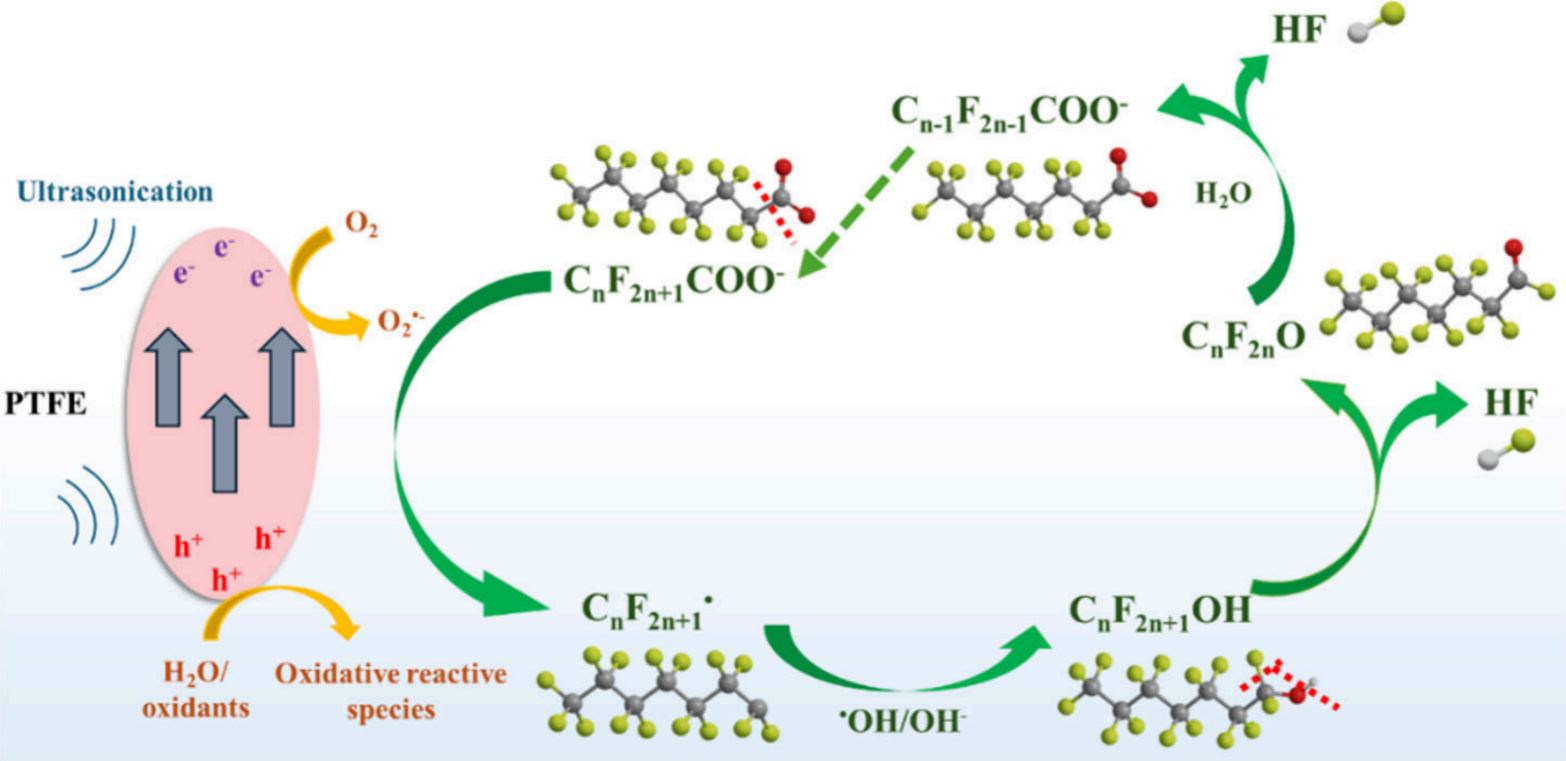
PFAS degradation pathway via oxidative chain-shortening by piezocatalysis.

Although PTFE serves as a novel piezocatalyst for PFAS degradation, it still faces significant drawbacks, primarily linked to the high energy demand and high interference from water matrices. First, ultrasonication is necessary for introducing surface defects in PTFE, leading to an intensive energy demand.^86,135,136^ To reduce energy demand, the use of alternative mechanical activation approaches could be investigated.^137^ In addition, further modification of PTFE to increase its sensitivity toward mechanical activation is recommended, which can enhance its catalytic performance while reducing energy demand. Moreover, complex water matrices can easily scavenge reactive species and absorb ultrasonic energy, further reducing the efficacy of the PTFE piezocatalyst.^135,137,138^ Subsequently, surface modifications on PTFE could further enhance its adaptability toward different substances in water.

### 3.4 Advanced Reduction Processes

Carbonaceous materials, notably indole-based materials, have also been applied in ARPs for PFAS degradation with reaction pathways different from those of conventional oxidative processes. ARPs involve the activation of UV light with certain chemical additives (e.g., sulfite, iodide and indole and its derivatives), where reductive reactive species (e.g., e_aq_^–^ and hydride radicals) are generated for reductive PFAS degradation.^18^ Within the reductive reactive species, e_aq_^–^ plays the key role in breaking the C–F bond due to its high reductive potential (−2.9 V vs SHE), higher than that of the C–F bond (2.7 V vs SHE).^139^ As illustrated in Figure 5, indole-based materials enable the efficient generation of e_aq_^–^ under UV irradiation and the generated e_aq_^–^ can directly cleave the robust C–F bonds in PFAS, inducing direct defluorination via H/F exchange.^18,140^ Subsequently, the products undergo further defluorination and chain-shortening via the DHEH pathway, leading to the formation of inorganic products and intermediates.^18^

**Figure 5:**
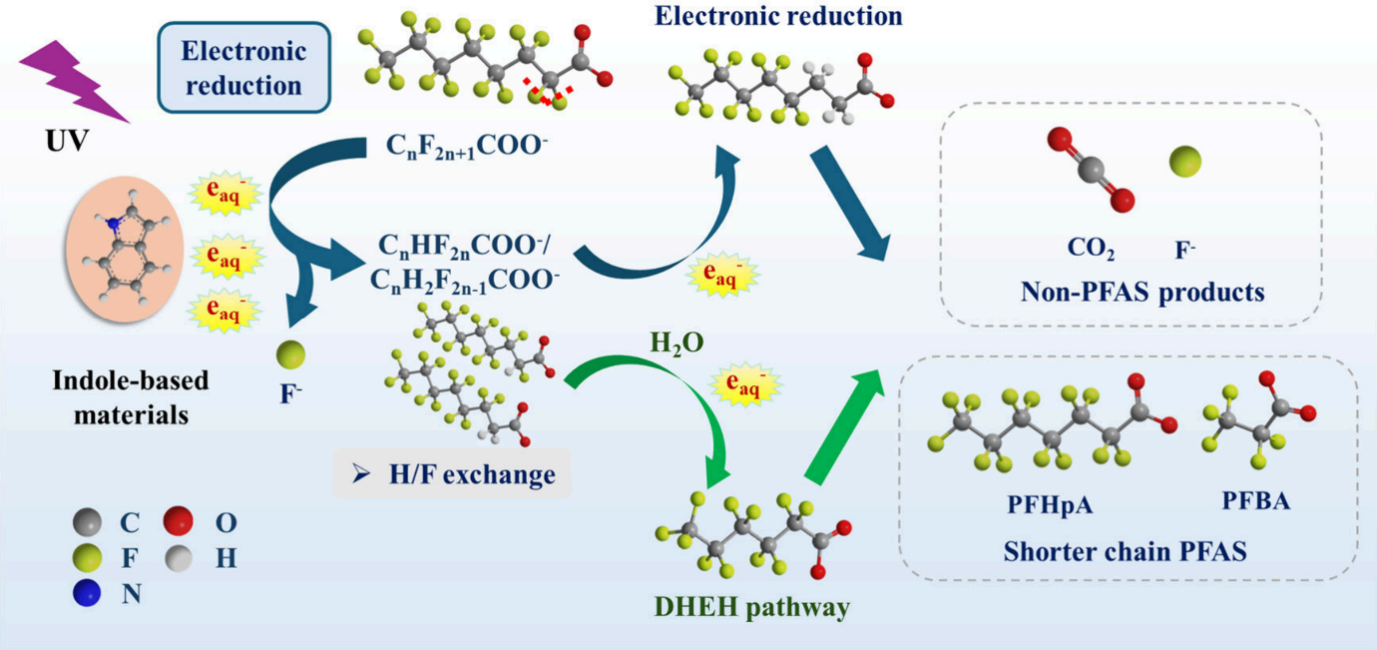
Degradation of PFAS by ARPs with indole-based materials via electron-induced reduction, followed by subsequent reductive defluorination and oxidative DHEH pathway.

As for the adsorptive role, indole-based materials concentrate PFAS near reactive sites to circumvent diffusion limitations for enhanced degradation. Adsorption arises via π–π interactions between the aromatic rings of indole and PFAS fluorinated chains, supplemented by hydrophobic associations and electrostatic interactions modulated by structural functionalization. Additionally, supramolecular indole assemblies (e.g., π–π stacked or hydrogen-bonded architectures) can act as molecular sieves, selectively adsorbing PFAS,^141^ creating localized high-concentration microenvironments. This proximity ensures that photogenerated e_aq_^–^ directly reduces the adsorbed PFAS, minimizing electron loss. For instance, indole-clay composites leverage the layered structure of montmorillonite to synergistically trap PFAS while enhancing electron transfer.^140,142^ It is notable that the adsorption ability is tunable, in which adding alkyl chains amplifies the hydrophobicity, while incorporating charged groups strengthens the ionic interactions with specific PFAS subclasses (e.g., PFCAs and PFSAs).^143^

Indole and its derivatives also serve a catalytic role in ARPs for PFAS degradation.^30,140^ For example, Chen et al. demonstrated that trace concentrations of indole (≤1 mM) degraded >90% of PFOA within 4 h under UV light, showcasing its catalytic efficiency.^87^ By embedding derivatives like 3-indole-acetic acid in organomontmorillonite clay, the indole radical cations can be stabilized on the clay surface, enhancing catalytic activity by prolonging electron lifetimes and improving electron transfer efficiency.^140,142^ Chemical modifications, such as introducing electron-donating groups, tailor indole derivatives to target specific PFAS subclasses, optimizing the reaction kinetics and selectivity.^30,141^ The use of indole-based materials in ARPs outperforms conventional electron donors (e.g., sulfite) due to lower activation energy, higher photostability, and tunable electronic properties.^25,87,141^

Despite their potential, the use of indole-based materials for ARPs faces several limitations. Disturbances of various water matrices, such as dissolved oxygen, ions, dissolved organic matter (DOM), and suspended solids, is a critical issue that significantly reduces PFAS degradation efficiency due to the quenching of e_aq_^–^ and blocking of the UV source.^18,87^ Hence, pretreatment of raw water (e.g., deoxygenation and filtration) can be integrated to reduce potential interference from water matrices. Moreover, indole-based materials are prone to chemical loss, which cannot be easily recovered postreaction, raising concerns that residual indole may accumulate in treated water and require costly replenishment. Thus, the immobilization of indole on certain substrates, forming a heterogeneous catalyst, can prevent its leaching into the environment. Collectively, these factors highlight the need for rigorous monitoring of reaction byproducts and utilization of indole-based material in heterogeneous ARPs systems to mitigate secondary pollution risks in homogeneous photocatalysis systems.

### 3.5 Thermal Treatment

Carbonaceous materials, mainly AC and biochar, have been employed to facilitate the thermal degradation of PFAS. This technology involves the application of high temperatures to overcome the energy barrier of the C–F bonds to induce cleavage, which, under suitable conditions, can ensure complete mineralization and defluorination of PFAS and avoid byproduct formation.^28,144^ Primarily, carbonaceous materials play two roles in thermal PFAS degradation: (i) concentrating PFAS by adsorption; and (ii) providing thermally stable platforms for decomposition.^27,36,145^ The thermal decomposition typically involves the homolytic cleavage of C–F bonds, forming perfluoroalkyl radicals that can further participate in chain reactions to generate various products.^145^ The formation of perfluoroalkyl radicals is crucial, as they can react with other species to facilitate the breakdown of PFAS into less harmful compounds. As such, oxidative environments (e.g., air) can enhance PFAS breakdown by introducing oxygen to react with perfluoroalkyl radicals, producing highly unstable perfluoroalkyl peroxy radicals that undergo further decomposition via β-scission or abstraction reactions to oxygen-containing products, such as tetrafluoroethene (C_2_F_4_), carbonyl fluoride (CF_2_O) and CO_2_, as shown in Figure 6.^146,147^ Additionally, oxygen also acts as an electron acceptor to facilitate PFAS mineralization, enabling complete oxidation pathways to convert organic fluorine into inorganic F^–^ ions and HF.^145,148^

**Figure 6:**
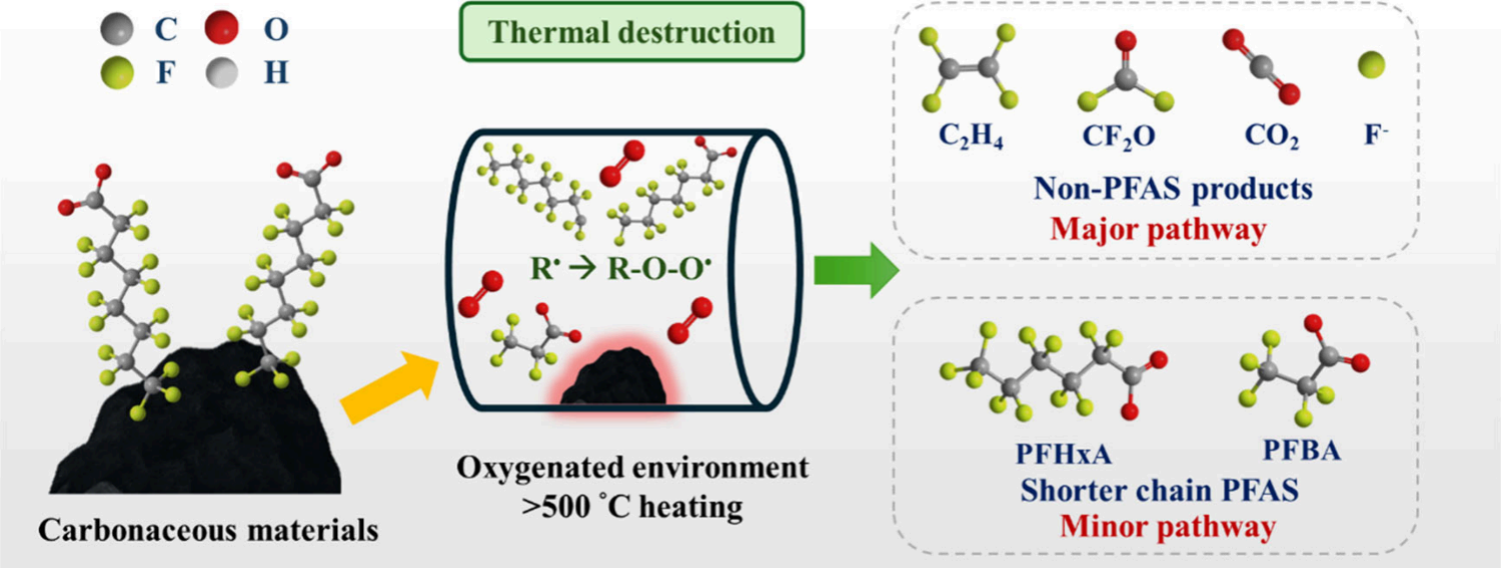
PFAS degradation pathways via thermal treatment under an oxygenated environment.

Kinetic analysis reveals that the multisteps during PFAS degradation, including adsorption, phase transitions, bond cleavage, and radical recombination, are influenced by the heating rate and energy input applied to the adsorbed PFAS molecules.^50,144^ Upon heating, PFAS undergo several phase transitions, starting with melting and evaporation, which, if not controlled, can hinder complete thermal decomposition.^144^ Rapid heating techniques, such as induction heating, have been explored to minimize the time PFAS spends in a melted state and avoid undesirable PFAS evaporation, enabling most of the adsorbed PFAS on carbonaceous materials to undergo thermal decomposition.^144^ This method ensures that thermal energy is applied uniformly, promoting simultaneous melting and degradation of PFAS, thus, reducing the formation of reactive intermediates.

Considering the adsorptive role of carbonaceous materials, their extensive pore network physically traps PFAS molecules, concentrating contaminants for subsequent degradation. For example, AC utilizes its well-developed micropores and mesopores network to adsorb PFAS, achieving high local contaminant concentrations.^27^ This adsorption capacity can possibly be regenerated through a two-stage thermal process: low-temperature drying prevents PFAS volatilization, while thermal treatment at >700 °C could reach high defluorination rate (>80%), with moderate (∼22%) loss in the SSA.^27,35,36^ The considerable SSA loss reduced the adsorption capacity and changed the morphology of AC,^149^ necessitating landfill disposal with environmental impact. Moreover, the strong affinity of PFAS to the nonpolar regions of carbonaceous materials is crucial for optimizing the degradation efficiency because such physical adsorption concentrates PFAS on the adsorbents’ surface, where thermal interactions occur.^50^ Such proximity facilitates the effective application of thermal energy, driving PFAS degradation. In contrast, biochar, derived from waste biomass, is regarded as a sustainable adsorbent. By leveraging its nonpolar regions and porous structure, biochar can adsorb PFAS while enabling localized thermal degradation.^150^ Although the adsorption kinetics and capacity of AC are several order-of-magnitude higher than that of biochar, thanks to its hierarchical pore network, the lower regeneration temperature (∼500 °C) of biochar with less intermediates generation makes it a thermodynamically favored option.^28,151^ Moreover, under low pyrolysis temperature, the adsorption capacity of the regenerated biochar outperforms pristine biochar, favoring the regeneration of biochar for further use as PFAS adsorbent.^152^

Modifications in carbonaceous materials can further enhance their catalytic roles, thus facilitating the degradation of PFAS at lower thermal activation energies. For instance, the incorporation of alkali and alkaline-earth metal additives to pristine AC enhances mineralization efficiency through inducing ionic exchanges to weaken the PFAS headgroup bonds, thus reducing the energy required to break the C–F bonds.^148^ At elevated temperatures, metal oxides generate basic sites on the surfaces of carbonaceous materials, stabilizing the transition states during radical reactions and propagating chain reactions during PFAS degradation.^148^ Additionally, metal fluorides are formed during heating the sequester fluoride ions, preventing the formation of harmful byproducts and enhancing defluorination. The catalytic process is further amplified by high temperatures, which drive the homolytic cleavage of PFAS functional groups, generating reactive radicals (e.g., perfluoroalkyl radicals) that initiate degradation pathways.^145,148^ This synergy between catalytic additives and carbonaceous supports enables efficient PFAS mineralization into less toxic organofluoride products.

Several unresolved drawbacks and knowledge gaps limit the application of AC and biochar to PFAS thermal degradation. One of the prior concern is the balance between the extensive pore structure and surface chemistry. While hierarchical micro/mesopores enhance PFAS adsorption and degradation by improving mass transfer and thermal exposure,^50^ excessive micropores lead to pore clogging during the thermal cycles. Surface functional groups, such as oxygenated sites, may weaken C–F bonds,^28^ but their instability under prolonged heating risks deactivation or toxic byproduct formation. Strategy of tailored pore architectures (e.g., graded micro/mesoporous gradients) could optimize PFAS accessibility while mitigating pore clogging, while approaches to stabilize oxygenated sites against degradation can be adopted to sustain catalytic activity without deactivation or risk of byproduct formation.

Another concern is that surface modifications aimed at boosting catalytic activity could inadvertently promote competitive adsorption of nontarget contaminants or generate harmful intermediates. Surface engineering to enhance PFAS selectivity could potentially suppress competitive adsorption of nontarget contaminants, while the use of metal-based additives favors complete mineralization while avoiding harmful intermediate formation. To guide future material modifications, deeper mechanistic insights into the interplay among PFAS molecular size, pore architecture, and reaction mechanisms in thermal treatment are needed. Addressing these issues is critical to advancing carbonaceous materials from laboratory-scale promise to applicable solutions.

## 4 Challenges and Future Perspectives

The advancement of carbonaceous materials for PFAS destruction is constrained by several critical challenges, mainly from material design, performance deterioration, toxicity issues related to material leaching and PFAS degradation intermediates, and scalability, as shown in Figure 7. Addressing these challenges requires a systematic re-evaluation of material stability, synthesis pathways, and operational frameworks to bridge the gap between laboratory innovation and real-world applications.

**Figure 7:**
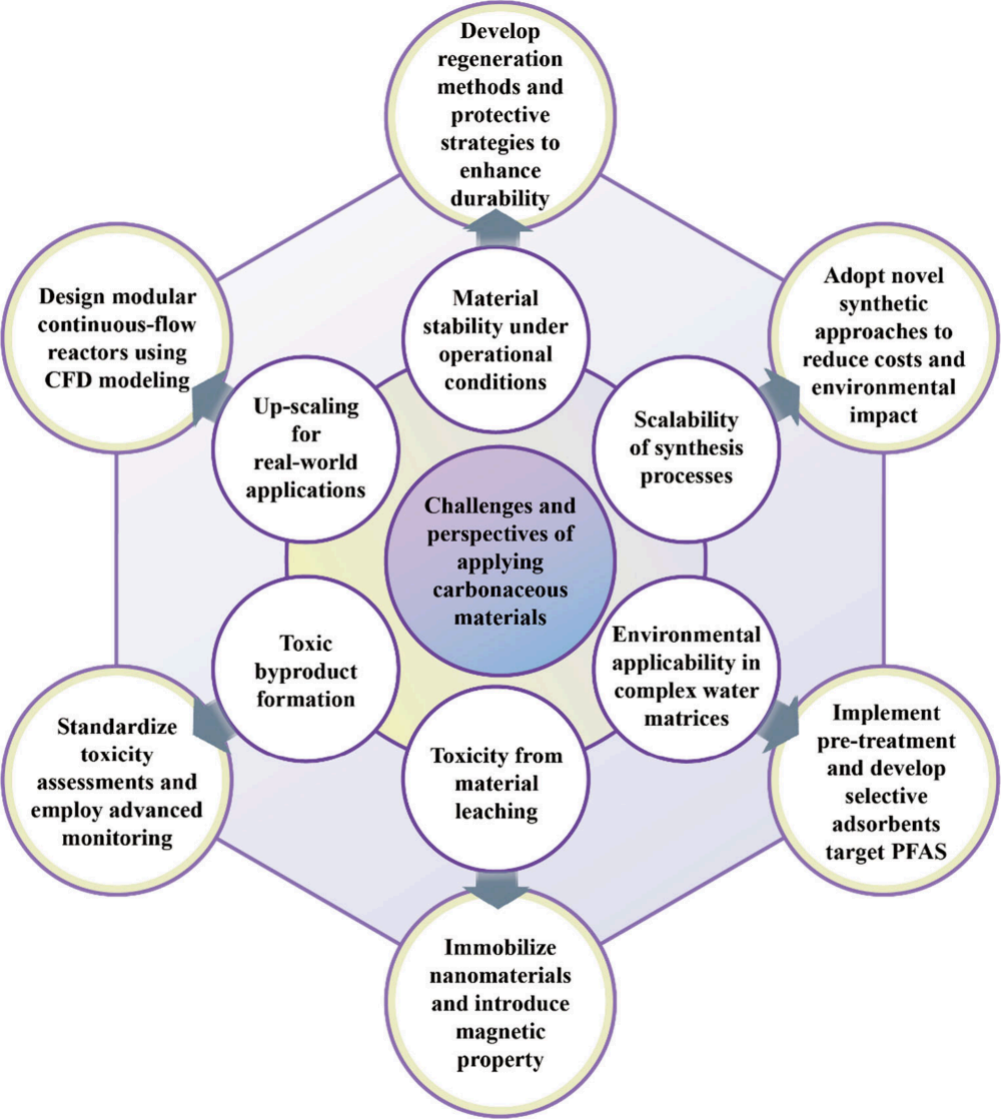
Challenges and perspectives of using carbonaceous materials for PFAS degradation.

### 4.1 Poor Stability and Scalability of Carbonaceous Materials

The advancement of carbonaceous materials for PFAS degradation presents critical challenges concerning their material stability and synthesis. A primary challenge is the long-term stability of these materials under operational conditions. For instance, in photocatalysis, the gradual degradation of photoactive sites and photocorrosion of photocatalysts under prolonged illumination reduce the treatment efficiency and material lifespan. Similarly, carbonaceous materials experience diminished performance following thermal treatment at extreme temperatures, due to structural deterioration that leads to the reduction in SSA,^27,153,154^ compromising their reusability. Hence, material stability must be improved through innovative design strategies, such as incorporating suitable and highly operable regeneration methods after prolonged operation, introducing protective heteroatom dopants, or developing robust hybrid architectures resistant to photocatalytic deactivation and thermal degradation.

Furthermore, synthesizing advanced materials like graphene composites and functionalized CNTs involves energy-intensive processes, lengthy reaction times, and hazardous chemicals, leading to high production costs and low yields that limit their scaling for potential commercialization^155,156^ Additionally, conventional synthesis (e.g., hydrothermal/solvothermal processes) methods are highly energy-intensive, consuming up to 149 MJ of energy and generating 12–86 kg of CO_2_-equivalent per kg of material, limiting their viability in sustainable water treatment.^157,158^ In addition, thermal treatment and regeneration of carbonaceous materials demand high temperatures, compounding the cumulative energy demand and greenhouse gas emissions over the lifecycle of the materials. To address these challenges, the synthesis processes require optimization to achieve energy savings, cost-effectiveness, and environmentally friendliness. Emerging techniques, including microwave-assisted synthesis, biomass-derived carbon fabrication, and mechanochemical processing, show potential for reducing energy consumption and eliminating toxic reagents. By overcoming these material science challenges, next-generation carbonaceous materials can enable the introduction of more efficient and sustainable solutions for PFAS degradation in water treatment applications.

### 4.2 Performance Deterioration Due to Low PFAS Concentration and Complex Water Matrices

Beyond engineering challenges, environmental variability further complicates the practical deployment of these technologies. Notably, PFAS concentrations in natural water bodies and wastewater streams (at ng/L to μg/L levels) are orders of magnitude lower than those tested in laboratories (mg/L level),^159^ leading to diffusion-limited reaction kinetics and reduced degradation rates.^160^ Subsequently, the aforementioned “concentrate-and-destroy” strategy serves as an outstanding solution, leveraging preconcentration techniques primarily via adsorption to amplify PFAS levels prior to degradation.

Moreover, complex water matrices containing suspended solids, DOM, and ionic species interfere with the treatment efficacy. For instance, suspended solids can obstruct light pathways in photocatalytic systems, while DOM competes with PFAS for adsorption sites and scavenging reactive species, diminishing removal efficiency.^94^ Thus, suitable pretreatment, such as sand filtration and sedimentation, should be incorporated to remove impurities that interfere with PFAS degradation. Concurrently, the development of carbonaceous materials with selective adsorption capabilities, such as molecularly imprinted polymers or size-exclusion pores, could mitigate matrix interference by preferentially targeting PFAS over competing impurities. Antifouling surface modifications, including hydrophilic coatings, may further enhance material durability in heterogeneous environments.

### 4.3 Toxicity Due to Material Leaching

The application of carbonaceous materials for PFAS degradation is hindered by toxicity risks from material leaching, posing threats to the environment and human safety. Potential leaching of nanomaterials such as CNT- and GO-based materials exhibits cytotoxic effects due to their nanoscale dimensions and high surface reactivity.^161^ These risks are exacerbated by the propensity of nanoparticles to migrate from treatment systems to water streams, posing secondary contamination hazards. Studies indicate that CNTs can induce oxidative stress and inflammatory responses in mammalian cells, while GO sheets may disrupt cellular membranes, raising concerns about their persistence in ecosystems.^161–163^ To mitigate leaching, extra considerations on material and system design, such as the immobilization of nanomaterials within macrostructured frameworks (e.g., 3D-printed monoliths or carbon aerogels), magnetic functionalization via the introduction of magnetic nanoparticles, and the inclusion of size-exclusion membranes at the outlet, should be addressed in future research to retain catalytic functionality while minimizing material leaching.

### 4.4 Inability in Short-Chain PFAS Degradation

As mentioned above, existing research on PFAS degradation has rarely achieved complete degradation and defluorination. Specifically, the C–C bond cleavage pathways of PFAS degradation lead to the formation of short-chain intermediates due to incomplete degradation,^164,165^ primarily attributable to their unfavorable interfacial thermodynamics and poor adsorption onto catalyst surfaces. Typically, short-chain PFAS (≤C6) exhibit exceptionally high hydration energies, resulting in a strong thermodynamic tendency to remain dissolved in the bulk aqueous phase rather than adsorbed onto the carbonaceous materials.^92,166^ Furthermore, the shortened perfluoroalkyl chains lower the hydrophobicity of the PFAS molecules, diminishing the hydrophobic interactions and surface affinity between the intermediates and materials and hindering their further degradation.^92,167^ Hence, overcoming these challenges remains vital for achieving complete mineralization and defluorination. Future research should focus on innovative material design to enhance adsorption kinetics for short-chain PFAS, potentially through tailored surface charges, functional groups, or hierarchical nanostructures that counteract the high hydration energies of short-chain PFAS.

### 4.5 Toxicity from Degradation Intermediates, Products, and Chemical Additives

Toxicity from PFAS degradation intermediates, products, and chemical additives poses threats to human health and ecosystems. which are more mobile and persistent in the environment than long-chain PFAS.^168,169^ It has been reported that various short-chain PFAS can induce organ-specific toxicities, including reduced immune response, liver effects, and potential developmental neurotoxicity, leading to widespread exposure and potential impacts on humans and wildlife. Furthermore, short-chain PFAS are resistant to common degradation methods, increasing their prevalence in natural water sources. After PFAS degradation, toxic byproducts could be potentially generated to cause secondary pollution. For instance, carbonyl fluoride (CF_2_O), a highly toxic phosgene-like gas, is often produced during the thermal treatment of PFAS. CF_2_O poses a severe acute impact upon inhalation and readily hydrolyzes to HF, creating a secondary risk from a corrosive acid gas. Concurrently, chemical additives used in degradation processes, such as chloride electrolytes in electrocatalysis, may yield toxic byproducts like chlorinated organics or perchlorate.^170,171^ These byproducts introduce secondary contamination risks, undermining the sustainability of PFAS degradation technologies.^172^ Given the potential risks due to byproduct formation, a dual approach focusing on toxicity analysis and risk management is essential. Comprehensive assessments, including *in vitro*/*in vivo* toxicity testing and life-cycle analysis, could be standardized to evaluate the ecotoxicological impacts of carbonaceous materials prior to field application. Additionally, advanced degradation pathway control, provided by *in situ* spectroscopy, can provide insights into degradation pathways, enabling adjustments for complete mineralization and defluorination, while minimizing hazardous byproducts.

### 4.6 Limited Expandability of PFAS Degradation Systems

Apart from the material-related challenges, the transition of PFAS degradation technologies from lab-scale studies to full-scale applications is hindered by challenges in system scalability, as well. While lab-scale systems demonstrate promising efficiency, upscaling introduces complexities, such as inadequate light penetration in photocatalytic setups, limited mass transfer between carbonaceous materials and PFAS in electrocatalysis, and suboptimal flow dynamics. These factors collectively reduce the degradation efficiency and increase energy consumption. Furthermore, the chemical and resource demands of large-scale operations, such as oxidants in AOPs, compromise the cost-effectiveness and sustainability, particularly in continuous-flow systems. To address these challenges, future research should prioritize the design of modular, continuous-flow reactors optimized through computational fluid dynamics modeling. Such systems would enhance mass transfer and light utilization while minimizing energy inputs. Additionally, coupling real-time monitoring systems with adaptive process controls would enable dynamic adjustments to operational parameters, ensuring consistent performance under varying conditions. Potential large-scale studies will also be critical to validate scalability, sustainability, and economic viability prior to full-scale implementation.

## 5 Practical Implications

In this Review, we have elucidated how the intrinsic properties of carbonaceous materials, including adsorption and catalytic abilities, illustrate their effectiveness across diverse degradation technologies. These attributes underscore the strong potential of carbonaceous materials as suitable candidates for PFAS degradation. However, there is still a long way ahead for current technologies to achieve efficient and complete mineralization and defluorination of PFAS. Moreover, current research encounters practical challenges, including difficulties in the mass production of materials, poor replication of real-world complexities, toxic byproduct formation, and limited system scalability. To bridge this gap, future efforts must focus on adopting novel synthetic approaches, utilizing real wastewater to reflect environmental realities, integrating material modifications and pretreatment processes to minimize the release of toxic byproducts, and designing scalable degradation systems. By alignment of research with these priorities, the transition of carbonaceous materials from promising laboratory candidates to practical solutions for PFAS degradation can be accelerated.

## Supplementary Material



## Notes

The Supporting Information includes . The Supporting Information is available free of charge at https://pubs.acs.org/doi/10.1021/acs.est.5c07297.

List of PFAS under the regulation of EU; summary of the carbonaceous materials used for photocatalytic and electrocatalytic degradation of PFAS (PDF)
