# Supplementary material for: Unraveling the Adsorptive/Catalytic Roles of Carbonaceous Materials in Per- and Polyfluoroalkyl Substance (PFAS) Degradation: Current Status and Perspectives
Source: Environ Sci Technol. 2025 Oct 1;59(40):21401–20. doi: 10.1021/acs.est.5c07297 (PMC12529960; doi:10.1021/acs.est.5c07297)
Supplement: Supplementary file 1 [file es5c07297_si_001.pdf]

Supplementary information for

Unraveling the adsorptive/catalytic roles of carbonaceous materials in per- and polyfluoroalkyl substances (PFAS) degradation: current status and perspectives

Justin H. K. Man [a 1], Zexiao Zheng [a 1], Xiaoying Wang [a], Howard Y. M. Cheung [a], Zibo Xu [a], Jonathan J. Calvillo Solís [a], *Irene M. C. Lo [a,b]

[a] Department of Civil and Environmental Engineering, The Hong Kong University of Science and Technology, Hong Kong, China

[b] Institute for Advanced Study, The Hong Kong University of Science and Technology, Hong Kong, China

* Corresponding author. Email address: cemclo@ust.hk; (Chair Professor Irene M. C. Lo); Tel.: +852 2358 7157; Fax: +852 2358 1534.

[1] These authors contributed equally to this work.

The supplementary materials include 23 Pages and 3 Tables.

Table S1. List of PFAS to be analyzed for the EU Drinking Water Directive

Type of PFAS	Abbreviation	Chemical structure
Perfluorobutanoic acid	PFBA	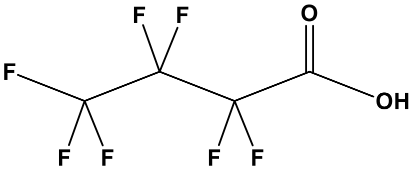
Perfluoropentanoic acid	PFPA	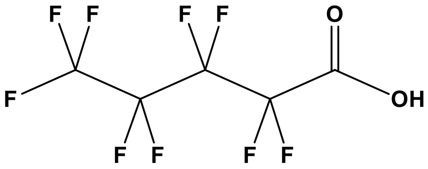
Perfluorohexanoic acid	PFHxA	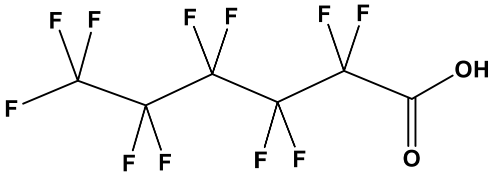
Perfluoroheptanoic acid	PFHpA	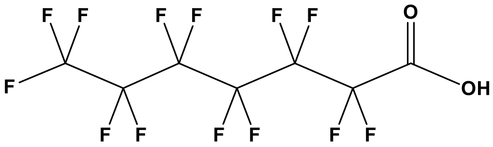
Perfluorooctanoic acid	PFOA	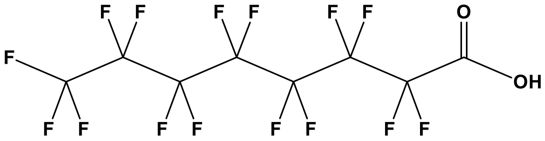
Perfluorononanoic acid	PFNA	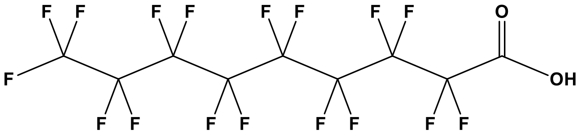
Perfluorodecanoic acid	PFDA	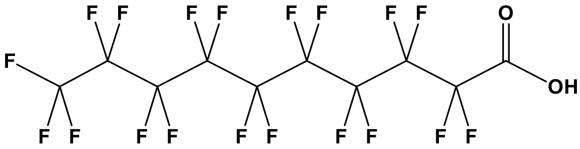
Perfluoroundecanoic acid	PFUnDA	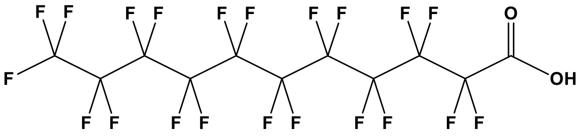

Perfluorododecanoic acid	PFDoDA	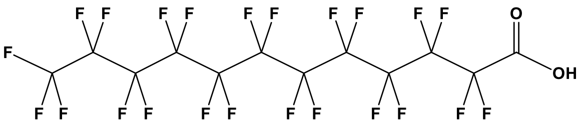
Perfluorotridecanoic acid	PFTrDA	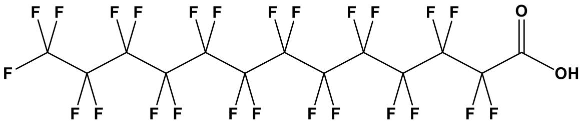
Perfluorobutane sulfonic acid	PFBS	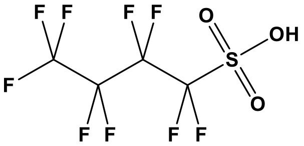
Perfluoropentane sulfonic acid	PFPS	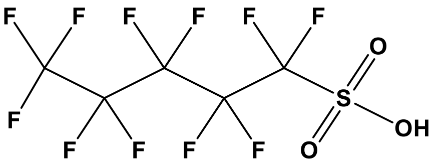
Perfluorohexane sulfonic acid	PFHxS	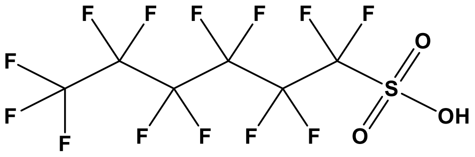
Perfluoroheptane sulfonic acid	PFHpS	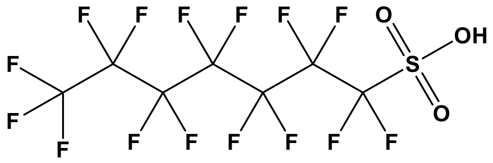
Perfluorooctane sulfonic acid	PFOS	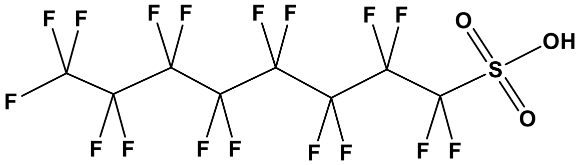
Perfluorononane sulfonic acid	PFNS	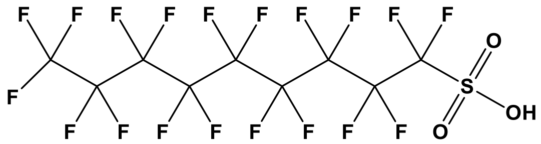
Perfluorodecane sulfonic acid	PFDS	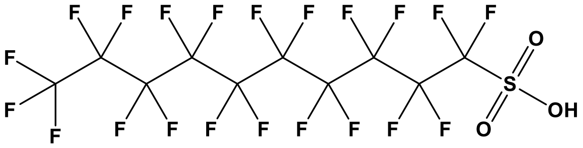

Perfluoroundecane sulfonic acid	-	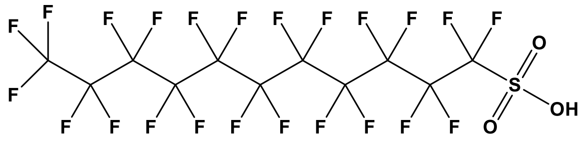
Perfluorododecane sulfonic acid	-	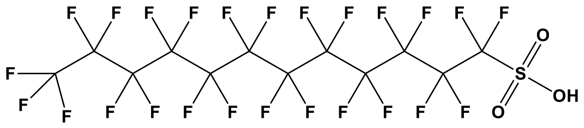
Perfluorotridecane sulfonic acid	-	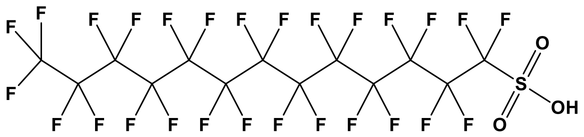

Table S2. Performance of photocatalytic systems using carbonaceous photocatalysts for PFAS degradation

Photocatalytic material	Experimental conditions	Type(s) of PFAS	Treatment duration	Removal efficiency	Defluorination rate (if applicable)	Key degradation mechanism(s)	Ref.
**Activated carbon (AC)-based materials**							
Fe/TNTs@AC	Adsorption:<br>$[PFOA]_0$ = 100 μg/L; $[material]_0$ = 1 g/L; pH = 7.0 ± 0.3; temperature = 25°C; dark condition<br><br>Photocatalysis:<br>$[material]_0$ = 4 g/L; pH = 7.0 ± 0.3; temperature = 25°C; 254 nm UV lamp	PFOA	Adsorption: 4 h<br>Photocatalysis: 4 h	Adsorption: >99% removal in 2 h<br>Photocatalysis: 91.3% of degradation in 4 h	62%	DHEH pathway	[1]
Ga/TNTs@AC	$[PFOS]_0$ = 100 μg/L; $[Photocatalyst]_0$ = 3 g/L; pH = 7.0 ± 0.1; temperature = 30 - 40°C, UV lamp with an intensity of 210 W/m^2	PFOS	4 h	75%	66.2%	DHEH pathway	[2]

Bi/TNTs@AC	Adsorption: [GenX]$_0$ = 100 μg/L; [material]$_0$ = 1 g/L; pH = 7.0 ± 0.1; temperature = 25°C; dark condition<br><br>Photocatalysis: [material]$_0$ = 1 g/L; pH = 7.0 ± 0.3; temperature = 35°C; 254 nm UV lamp	GenX	Adsorption: 2 h<br>Photocatalysis: 4 h	Adsorption: >99% removal in 1 h<br>Photocatalysis: 70.0% of degradaion in 4 h	42.7%	Oxidative pathway (including DHEH pathway)	3
In/TNTs@AC	Adsorption: [PFOA]$_0$ = 100 μg/L; [material]$_0$ = 1 g/L; pH = 7.0 ± 0.3; temperature = 25°C; dark condition<br><br>Photocatalysis: [PFOA]$_0$ = 400 μg/L; [material]$_0$ = 4 g/L; pH = 7.0 ± 0.3; temperature = 25°C; 254 nm UV lamp	PFOA	Adsorption: 4 h<br>Photocatalysis: 4 h	Adsorption: >99% removal in 30 min<br>Photocatalysis: >99% of degradation in 4 h	~60%	DHEH pathway & In-facilitated redox cycle	4
**Graphene-based materials**							
In_2O_3-Graphene	[PFOA]$_0$ = 30 mg/L; [Photocatalyst]$_0$ = 0.5 g/L; room temperature, 15 W low-pressure Hg lamp	PFOA	3 h	100%	60.9%	Oxidative pathway	5

SiC/Graphene	$[PFOA]_0$ = 0.12 mmol/L; $[Photocatalyst]_0$ = 0.5 g/L; pH = 7; temperature = 25 ± 1°C, 254 nm UV lamp	PFOA	8 h	58.5%	N/A	DHEH pathway & Hydrodefluorination process via the Si–H/C–F redistribution	6
SiC/GQDs	$[PFOS]_0$ = 0.019 mmol/L; $[Photocatalyst]_0$ = 0.5 g/L; pH = 7; temperature = 25 ± 1°C, 254 nm UV lamp	Technical-PFOS containing 2-CF_3-PFOS, 6-CF_3-PFOS and linear-PFOS	20 h	2-CF_3-PFOS: 93.9% 6-CF_3-PFOS: 90.4% Linear-PFOS: 88.5%	N/A	DHEH pathway & Hydrodefluorination process via the Si–H/C–F redistribution	7
ZnO/rGO	$[PFOA]_0$ = 100 mg/L; $[Photocatalyst]_0$ = 1 g/L; pH = 7.0, Solar irradiation at 11:00 am to 2:00 pm	PFOA	1 h	90.91%	N/A	N/A	8
TiO_2-rGO	$[PFOA]_0$ = 0.24 mmol/L; $[Photocatalyst]_0$ = 0.1 g/L; pH = 3.8; temperature = 20 - 25°C, 150 W medium-pressure Hg lamp	PFOA	12 h	93 ± 7%	N/A	Oxidative pathway	9
Pb-$BiFeO_3$/rGO	$[PFOA]_0$ = 50 mg/L; $[Photocatalyst]_0$ = 0.1 g/L; pH = 2.0; room temperature, 5 W 254 nm UV lamp	PFOA	8 h	48.0%	37.6%	DHEH pathway	10

TiO_2-rGO	$[PFOA]_0$ = 0.24 mmol/L; $[Photocatalyst]_0$ = 0.1 g/L; pH = 3.8; temperature = 25°C, 150 W medium-pressure Hg lamp	PFOA	8 h	86%	30%	Oxidative pathway	11
3D SG-TiO_2 QD aerogel	$[PFOA]_0$ = 0.3 mmol/L; $[Photocatalyst]_0$ = 0.1 g/L; pH = 2.0; room temperature, 5 W 254 nm UV lamp	PFOA	10 h	1.898 E^{-4}/s	~28%	DHEH pathway	12
**Other carbonaceous materials**							
TiO_2–MWCNT	$[PFOA]_0$ = 30 mg/L; $[photocatalyst]_0$ = 1.6 g/L; pH = 5.0; temperature = 23 ± 3 °C; 300 W medium-pressure Hg lamp	PFOA	8 h	94%	N/A	DHEH pathway	13
BiOHP/CS	$[PFOA]_0$ = 200 μg/L; $[photocatalyst]_0$ = 1 g/L; pH = 7.0; 18 W low-pressure Hg lamp	PFOA	Adsorption: 2 h Photocatalysis: 4 h	>90% degradation in 4 h	32.5%	DHEH pathway	14
FeO/CS	$[PFOA]_0$ = 200 μg/L; $[photocatalyst]_0$ = 1 g/L; pH = 7.0; simulated solar light	PFOA	Adsorption: 1 h Photocatalysis: 4 h	95.2%	57.2%	DHEH pathway	15

**MOF-based materials**							
Lignin/PVA/bi-MOF membrane	$[PFOA]_0$ = 20 mg/L; $[PMS]_0$ = 8 mM; pH = 7.0, Solar irradiation at 11:00 am to 2:00 pm	PFOA	3 h	89.6%	49%	DHEH pathway	16
F-TiO_2@MIL-125	$[PFOA]_0$ = 300 μmol/L; $[MOF]_0$ = 0.02 g/L; temperature = 25 °C, 150 W medium-pressure Hg lamp	PFOA	10 h	1.221 E^{-4}/s	N/A	DHEH pathway	17
In-MOF/BiOF	$[PFOA]_0$ = 15 mg/L; $[MOF]_0$ = 0.5 g/L; room temperature, Solar irradiation at 11:00 am to 2:00 pm	PFOA	3 h	100% in 150 min	34%	Oxidative pathway	18
MIL-125-NH_2 (Ti-based MOF)	$[PFOA]_0$ = 100 μg/L; $[MOF]_0$ = 2.5 g/L; [glucose] = 0.5 M; temperature = 20 °C, 450 W medium-pressure Hg lamp	PFOA	24 h	100%	66.7%	DHEH pathway & H/F exchange	19
MIL-177-HT (Ti-based MOF)	$[PFOA]_0$ = 100 μg/L; $[MOF]_0$ = 2.5 g/L; [TEOA] = 2.5% (v/v); temperature = 20 °C, 450 W medium-pressure Hg lamp	PFOA	24 h	83.0%	32%	DHEH pathway & H/F exchange	20
FeOCl/(Bi-MOF-BiOCl)$_{100}$	$[PFOA]_0$ = 50 mg/L; $[MOF]_0$ = 75 g/L; pH = 5.85; 253.7 nm UV lamp	PFOA & HFPO-TA (C7)	4 h	PFOA: >99.9% in 30 min<br>HFPO-TA: 83.9% in 4 h	PFOA: 64.3%<br>HFPO-TA: 43.2%	DHEH pathway & H/F exchange	21

**Carbon nitride (C_3N_4)-based materials**							
$ZIF67@C_3N_4$<br>MIL-100(Fe)@C_3N_4	$[PFOA]_0$ = 50 μg/L; $[photocatalyst]_0$ = 1 g/L; 300 W iodine-tungsten lamp	PFOA	Adsorption: 4 h<br>Photocatalysis: 8 h	79.2%<br>60.5%	N/A	Oxidative pathway	22
Exfoliated C_3N_4 with Fe^{3+}	$[PFOA]_0$ = 500 μg/L; $[photocatalyst]_0$ = 2 g/L; $[Fe^{3+}]_0$ = 0.005 mol/L; pH 2.5; temperature = 25 ± 3 °C; 12W low-pressure UV lamp and 30 W LED lamp as visible light source	PFOA	UV: 19 h<br>Visible: 70 h	UV: >95% in 19 h<br>Visible: 99% in 70 h	UV: >95% in 19 h<br>Visible: >95% in 70 h	DHEH pathway	23

Table S3. Performance of photocatalytic systems using carbonaceous photocatalysts for PFAS degradation

Anodic material	Cathodic material	Applied current density/ external bias	Electrolyte	Other experimental conditions	Type(s) of PFAS	Treatment duration	Removal efficiency	Defluorination rate (if applicable)	Key degradation mechanism(s)	Ref.
**Carbonaceous anode used**										
Boron-doped reduced graphene oxide (BRGO)–coated sponge electrode	Stainless steel sponge	23 mA/cm^2	10 mM phosphate buffer	$[PFOA]_0$ = 200 μg/L; temperature = 25 ± 1 °C; flow-through cylindrical reactor; flow rate = 2.5 - 10 mL/min	PFOS, PFOA, PFHxS, PFHxA, PFBS, PFBA	30 bed volumes	PFOS: 67 ± 4.1%<br>PFOA: 43.6 ± 12.1%<br>PFHxS: 31.3 ± 2.9 %<br>PFHxA: 23.5 ± 2.1%<br>PFBS: 26.7 ± 1.2%<br>PFBA: 16.6 ± 1.2 %	PFOS: 67 ± 4.1%<br>PFOA: 43.6 ± 12.1 %<br>PFHxS: 31.3 ± 2.9 %<br>PFHxA: 23.5 ± 2.1 %<br>PFBS: 26.7 ± 1.2 %<br>PFBA: 16.6 ± 1.2 %	Anodic oxidation	24
N-doped graphene oxide nanosheet/CeO_2 @Ti_4O_7 membrane	Stainless steel electrode	20 mA/cm^2	0.1 mol/L $NaClO_4$	$[GenX]_0$ = 10 mg/L in batch, 2 μmol/L in flow-through; room temperature; two-electrode configuration for batch tests, flow-through REM reactor for flow-through tests	GenX	Batch: 2 h<br>Flow-through: 4 h	Batch: 97%<br>Flow-through: up to >95% in single pass at low flux (200 L m^{-2} h^{-1})	70 - 90% in real fluorochemical wastewater	Anodic oxidation	25
Ti/BDD electrode	Ti plate	10 mA/cm^2	10 mmol/L $NaClO_4$	$[PFAS]_0$ = 0.25 mmol/L; pH = 3; temperature = 25 ± 1 °C; stirring speed = 800 rpm	PFNA, PFDA	3 h	PFNA: 98.7 ± 0.4%<br>PFDA: 96.0 ± 1.4%	PFNA: 88.0 ± 1.5%<br>PFDA: 50.7 ± 1.5%	Anodic oxidation	26

B/N codoped diamond/Ti electrode	Pt foil	4 mA/cm^2	0.05 mol/L Na_2SO_4	$[PFOA]_0$ = 50 mg/L; Initial pH = 4.8; H-cell with three-electrode configuration	PFOA	3 h	99.3% in 1.5 h	76.8% in 3 h	Anodic oxidation	27
BDD electrode	Stainless steel electrode	50 mA/cm^2	Real groundwater and landfill leachate	$[PFAS]_0$ = 3.1 ± 0.4 µg/L in groundwater, 2.2 ± 0.2 µg/L in leachate, up to 19 µg/L in foam; pH = ~ 7.5 - 7.8; 20 L flow-through electrochemical cell	29 PFAS including PFOS, PFOA, PFBS, PFHxS, PFBA, PFPeA, PFHxA, PFHpA, etc.	9 h	Groundwater & leachate: up to 84% Foam: ~65%	N/A	Anodic oxidation	28
Si/BDD electrode	Ti plate	1.8 - 40 mA/cm^2 5 - 50 V	10 mmol/L Na_2SO_4 and 2 mmol/L NaCl	$[PFAS]_0$ = 1 mg/L; Initial pH = 7; temperature = 25 ± 1 °C; laboratory-scale undivided batch electrochemical cel	PFOA, PFOS, PFHxS	5 h	PFOA: 94.0% PFOS: 89.1% PFHxS: 88.1%	N/A	Anodic oxidation	29
B/N-doped diamond (BND) electrode	Stainless steel mesh	5 mA/cm^2	0.1 mol/L Na_2SO_4	$[PFOA]_0$ = 50 mg/L; Initial pH = 6.5; flow-through cell with recirculation; conductivity = 15.8 mS/cm	PFOA	2 h	97 - 99% in 1 h	N/A	Anodic oxidation	30

**Carbonaceous cathode used**										
Pt sheet	GAC in stainless steel mesh	10 V	0.1 mol/L Na_2SO_4	$[PFAS]_0$ = 0.1 mmol/L; single-cell and H-cell configurations; N_2 purging	PFMeUPA & PFOS	PFMeUPA: 8 h	PFMeUPA: 100% in 8 h	PFMeUPA: 100% at 85°C PFOS: 3.6%	Cathodic reduction	31
Pt electrode	Hierarchically porous carbon (HPC) coated on carbon paper	-0.4 V	0.05 mol/L Na_2SO_4 + $FeSO_4$	$[PFOA]_0$ = 50 mg/L; pH = 2 - 6; one-compartment batch cell	PFOA	4 h	94.3% in 3 h	64.8% in 4 h	Electro-Fenton oxidation	32
Pt electrode	MOFs/carbon nanofibers composite membrane (MOFs/CNF)	-0.6 V vs. SCE	0.05 mol/L Na_2SO_4	$[PFOA]_0$ = 20 mg/L; pH = 3; Solar light source (20 - 35 mW/cm^2); Rectangular single-compartment cell; O_2 purging	PFOA	2 h	99%	59%	Photo-electro-Fenton oxidation	33
Pt sheet	Carbon paper modified with ZIF-67 and carbon black	-1.2 V vs. Ag/AgCl	50 mmol/L phosphate buffer	$[TFMAA]_0$ = 5 mg/L; temperature = 30 °C; two-compartment glass cell separated by cation exchange membrane; stirring speed = 250 rpm; N_2 purging	2-(trifluoromethyl) acrylic acid (TFMAA)	48 h	99.66%	97.16%	Cathodic reduction	34

**Both carbonaceous anode and cathode used**										
BDD electrode	PTMA-co-PTMPMA redox-copolymer functionalized electrode	Adsorption: 0.8 V vs. Ag/AgCl Desorption: -1.0 V vs. Ag/AgCl Defluorination: 10 mA/cm^2	20 mmol/L NaCl	$[GenX]_0$ = 0.1 mmol/L; room temperature; three-electrode configuration	GenX	Adsorption: 30 min Defluorination: up to 24 h	>95% of GenX adsorption in 9 min	100% in 24 h	Anodic oxidation & cathodic reduction	35
Si/BDD electrode	Si/BDD electrode	25 mA/cm^2	400 mg/L Na_2SO_4	$[PFAS]_0$ = 2 μM; pH = 5; room temperature; stirring speed = 250 rpm	Primarily PFOA	1 h	~90%	37%	Anodic oxidation	36
AC cloth for electrosorption; BDD electrode for defluorination	AC cloth	Redox-polymer electrodialysis: 1.0 V; Electrocatalysis: 20 mA/cm^2	10 - 20 mmol/L NaCl	$[PFAS]_0$ = 1 μmmol/L - 1 mmol/L; neutral pH; PFAS removal/desalination in closed loop at 10 mL/min; NF (1 kDa MWCO cellulose) and CEM membranes used	PFOA, PFHxA, PFBA, PFPrA, TFA	6 - 24 h	PFOA: ~90% PFHxA: 100% in 8 h PFBA: 90% by electrodialysis PFPrA: 100% in 6 h TFA: 100% in 6 h	PFOA: ~70% PFHxA: 76% PFPrA: 100% TFA: 100%	Anodic oxidation	37

Nb-supported-polycrystalline BDD electrode	Nb-supported-polycrystalline BDD electrode	10 and 50 mA/cm^2	Landfill leachate	Initial PFAS concentration = 51500 ± 3300 ng/L; conductivity = 20.7 ± 0.2 mS/cm; H-cell with three-electrode configuration for batch tests; 2 L/min recirculation for flow-through tests, with 3 anodes and 2 cathodes	Mixtures of various PFAS: C4 - C10 for PFCAs C3 - C8 for PFSAs	8 - 32 h	~80% total PFAS degradation in 32 h	N/A	Anodic oxidation	38
BDD electrode	Graphene-coated Ni foam	16 mA/cm^2	0.05 mol/L K_2SO_4	$[GenX]_0$ = 0.25 mmol/L; pH = 3; 80 mL undivided electrolytic cell	GenX	6 h	92.2 ± 1.0% TOC removal	89 ± 4.0% fluoride ion recovery	Electro-Fenton oxidation	39
BDD electrode	Stainless steel mesh	25 and 75 mA/cm^2	100 mM phosphate buffer (PB) or landfill leachate (LL) diluted 1:1 with water	$[PFAS]_0$ = 0.1 mg/L; pH = ~ 7.03 - 7.29; temperature = 20–25°C; 400 mL undivided electrolytic cell	PFOA & PFOS	8 h	PFOA: up to 99.9% in PB; up to 81% in LL PFOS: up to 84% in PB ; up to 80% in LL	N/A	Anodic oxidation	40

BDD electrode	BDD electrode	21.4 mA/cm^2	1.5 g/L Na_2SO_4	$[PFOA]_0$ = 10 mg/L in synthetic water; $[Total\ PFAS]_0$ = 40.6 - 53.2 µg/L; 200 mL solution in 300 mL beaker for batch tests; Flow rate = 1.13 mL/min with recirculation	Synthetic water: PFOA Wastewater: 11 PFAS including PFBA, PFPeA, PFHxA, PFHpA, PFOA, PFNA, PFDA, PFBS, PFHxS, PFOS, 6:2 FTSA	4 h		PFOA in synthetic water: 99.5% Wastewater: PFOA: 44–70% PFOS: 65–80% 6:2 FTSA: 42–52% ΣTotal PFAS: 56%	50% for PFOA in synthetic water	Anodic oxidation	41

References

(1) Li, F.; Wei, Z.; He, K.; Blaney, L.; Cheng, X.; Xu, T.; Liu, W.; Zhao, D. A Concentrate-and-Destroy Technique for Degradation of Perfluorooctanoic Acid in Water Using a New Adsorptive Photocatalyst. *Water Res.* **2020**, *185*, 116219. https://doi.org/10.1016/j.watres.2020.116219.

(2) Zhu, Y.; Xu, T.; Zhao, D.; Li, F.; Liu, W.; Wang, B.; An, B. Adsorption and Solid-Phase Photocatalytic Degradation of Perfluorooctane Sulfonate in Water Using Gallium-Doped Carbon-Modified Titanate Nanotubes. *Chem. Eng. J.* **2021**, *421*, 129676. https://doi.org/10.1016/j.cej.2021.129676.

(3) Zhu, Y.; Ji, H.; He, K.; Blaney, L.; Xu, T.; Zhao, D. Photocatalytic Degradation of GenX in Water Using a New Adsorptive Photocatalyst. *Water Res.* **2022**, *220*, 118650. https://doi.org/10.1016/j.watres.2022.118650.

(4) Arana Juve, J.-M.; Li, F.; Zhu, Y.; Liu, W.; Ottosen, L. D. M.; Zhao, D.; Wei, Z. Concentrate and Degrade PFOA with a Photo-Regenerable Composite of In-Doped TNTs@AC. *Chemosphere* **2022**, *300*, 134495. https://doi.org/10.1016/j.chemosphere.2022.134495.

(5) Li, Z.; Zhang, P.; Li, J.; Shao, T.; Jin, L. Synthesis of In_2O_3-Graphene Composites and Their Photocatalytic Performance towards Perfluorooctanoic Acid Decomposition. *J. Photochem. Photobiol. Chem.* **2013**, *271*, 111–116. https://doi.org/10.1016/j.jphotochem.2013.08.012.

(6) Huang, D.; Yin, L.; Niu, J. Photoinduced Hydrodefluorination Mechanisms of Perfluorooctanoic Acid by the SiC/Graphene Catalyst. *Environ. Sci. Technol.* **2016**, *50* (11), 5857–5863. https://doi.org/10.1021/acs.est.6b00652.

(7) Huang, D.; Yin, L.; Lu, X.; Lin, S.; Niu, Z.; Niu, J. Directional Electron Transfer Mechanisms with Graphene Quantum Dots as the Electron Donor for Photodecomposition of Perfluorooctane Sulfonate. *Chem. Eng. J.* **2017**, *323*, 406–414. https://doi.org/10.1016/j.cej.2017.04.124.

(8) Ong, C. B.; Mohammad, A. W.; Ng, L. Y.; Mahmoudi, E.; Azizkhani, S.; Hayati

Hairom, N. H. Solar Photocatalytic and Surface Enhancement of ZnO/rGO Nanocomposite: Degradation of Perfluorooctanoic Acid and Dye. *Process Saf. Environ. Prot.* **2017**, *112*, 298–307. https://doi.org/10.1016/j.psep.2017.04.031.

(9) Gomez-Ruiz, B.; Ribao, P.; Diban, N.; Rivero, M. J.; Ortiz, I.; Urtiaga, A. Photocatalytic Degradation and Mineralization of Perfluorooctanoic Acid (PFOA) Using a Composite TiO_2−rGO Catalyst. *J. Hazard. Mater.* **2018**, *344*, 950–957. https://doi.org/10.1016/j.jhazmat.2017.11.048.

(10) Shang, E.; Li, Y.; Niu, J.; Li, S.; Zhang, G.; Wang, X. Photocatalytic Degradation of Perfluorooctanoic Acid over Pb-$BiFeO_3$/rGO Catalyst: Kinetics and Mechanism. *Chemosphere* **2018**, *211*, 34–43. https://doi.org/10.1016/j.chemosphere.2018.07.130.

(11) Rivero, M. J.; Ribao, P.; Gomez-Ruiz, B.; Urtiaga, A.; Ortiz, I. Comparative Performance of TiO_2-rGO Photocatalyst in the Degradation of Dichloroacetic and Perfluorooctanoic Acids. *Sep. Purif. Technol.* **2020**, *240*, 116637. https://doi.org/10.1016/j.seppur.2020.116637.

(12) Zhu, C.; Xu, J.; Song, S.; Wang, J.; Li, Y.; Liu, R.; Shen, Y. TiO_2 Quantum Dots Loaded Sulfonated Graphene Aerogel for Effective Adsorption-Photocatalysis of PFOA. *Sci. Total Environ.* **2020**, *698*, 134275. https://doi.org/10.1016/j.scitotenv.2019.134275.

(13) Song, C.; Chen, P.; Wang, C.; Zhu, L. Photodegradation of Perfluorooctanoic Acid by Synthesized TiO_2–MWCNT Composites under 365 Nm UV Irradiation. *Chemosphere* **2012**, *86* (8), 853–859. https://doi.org/10.1016/j.chemosphere.2011.11.034.

(14) Xu, T.; Zhu, Y.; Duan, J.; Xia, Y.; Tong, T.; Zhang, L.; Zhao, D. Enhanced Photocatalytic Degradation of Perfluorooctanoic Acid Using Carbon-Modified Bismuth Phosphate Composite: Effectiveness, Material Synergy and Roles of Carbon. *Chem. Eng. J.* **2020**, *395*, 124991. https://doi.org/10.1016/j.cej.2020.124991.

(15) Xu, T.; Ji, H.; Gu, Y.; Tong, T.; Xia, Y.; Zhang, L.; Zhao, D. Enhanced Adsorption

and Photocatalytic Degradation of Perfluorooctanoic Acid in Water Using Iron (Hydr)Oxides/Carbon Sphere Composite. *Chem. Eng. J.* **2020**, *388*, 124230. https://doi.org/10.1016/j.cej.2020.124230.

(16) Hou, C.; Chen, W.; Fu, L.; Zhang, S.; Liang, C.; Wang, Y. Efficient Degradation of Perfluorooctanoic Acid by Electrospun Lignin-Based Bimetallic MOFs Nanofibers Composite Membranes with Peroxymonosulfate under Solar Light Irradiation. *Int. J. Biol. Macromol.* **2021**, *174*, 319–329. https://doi.org/10.1016/j.ijbiomac.2021.01.184.

(17) Kong, Z.; Lu, L.; Zhu, C.; Xu, J.; Fang, Q.; Liu, R.; Shen, Y. Enhanced Adsorption and Photocatalytic Removal of PFOA from Water by F-Functionalized MOF with in-Situ-Growth TiO_2: Regulation of Electron Density and Bandgap. *Sep. Purif. Technol.* **2022**, *297*, 121449. https://doi.org/10.1016/j.seppur.2022.121449.

(18) Wang, J.; Cao, C.-S.; Wang, J.; Zhang, Y.; Zhu, L. Insights into Highly Efficient Photodegradation of Poly/Perfluoroalkyl Substances by In-MOF/BiOF Heterojunctions: Built-in Electric Field and Strong Surface Adsorption. *Appl. Catal. B Environ.* **2022**, *304*, 121013. https://doi.org/10.1016/j.apcatb.2021.121013.

(19) Wen, Y.; Rentería-Gómez, Á.; Day, G. S.; Smith, M. F.; Yan, T.-H.; Ozdemir, R. O. K.; Gutierrez, O.; Sharma, V. K.; Ma, X.; Zhou, H.-C. Integrated Photocatalytic Reduction and Oxidation of Perfluorooctanoic Acid by Metal–Organic Frameworks: Key Insights into the Degradation Mechanisms. *J. Am. Chem. Soc.* **2022**, *144* (26), 11840–11850. https://doi.org/10.1021/jacs.2c04341.

(20) Wen, Y.; Kirchon, A.; Day, G. S.; Lin, H.; Smith, M. F.; Boehme, A.; Ozdemir, R. O. K.; Sharma, V. K.; Ma, X.; Zhou, H.-C. Photocatalytic Degradation of Perfluorooctanoic Acid (PFOA) by Metal Organic Framework MIL-177-HT: New Insights into the Role of Specific Surface Area, Charge Separation and Dimensionality. *Sep. Purif. Technol.* **2025**, *354*, 128877. https://doi.org/10.1016/j.seppur.2024.128877.

(21) Gu, M.; Duan, L.; Zhang, Z.; Jiang, Y.; Zhang, Q.; Zhou, L.; Yu, G.; Huang, J.

Multi-Charge Bridge Transfer Guiding Design of Photocatalyst with Oxygen Defects for Effective Degradation of PFAS in Fluoropolymer Production Wastewater. *Chem. Eng. J.* **2025**, *505*, 159124. https://doi.org/10.1016/j.cej.2024.159124.

(22) Su, P.; Zhang, C.; Liu, Y.; Zhang, J.; Djellabi, R.; Wang, R.; Guo, J.; Zhang, R.; Guo, H.; Ding, X.; Liu, X. Boosting PFOA Photocatalytic Removal from Water Using Highly Adsorptive and Sunlight-Responsive ZIF67/MIL-100(Fe) Modified C_3N_4. *J. Environ. Chem. Eng.* **2023**, *11* (5), 110765. https://doi.org/10.1016/j.jece.2023.110765.

(23) Li, J.; Liu, Y.; Song, Y.; Cao, L.; Dou, Y.; Yu, J.; Zhang, Y.; He, J.; Dai, W.; Yao, C.; Kong, D. Enhanced and Accelerated Degradation of PFOA Using Visible Light—Applying Semiconductor Carbon Nitride as an Accelerator. *J. Environ. Chem. Eng.* **2024**, *12* (1), 111653. https://doi.org/10.1016/j.jece.2023.111653.

(24) Duinslaeger, N.; Radjenovic, J. Electrochemical Degradation of Per- and Polyfluoroalkyl Substances (PFAS) Using Low-Cost Graphene Sponge Electrodes. *Water Res.* **2022**, *213*, 118148. https://doi.org/10.1016/j.watres.2022.118148.

(25) Liang, Y.; Wang, A.; Liang, S.; Sun, K.; Xie, R.; Zheng, C.; Zhang, S.; Tang, C.; Cheng, D.; Wang, J.; Huang, Q.; Lin, H. Durable Ti_4O_7 Heterojunction Composite Membrane Encapsulating N-Doped Graphene Nanosheets for Efficient Electro-Oxidation of GenX and Other PFAS in Fluorochemical Wastewater. *Environ. Sci. Technol.* **2025**, *59* (9), 4745–4755. https://doi.org/10.1021/acs.est.4c09423.

(26) Lin, H.; Niu, J.; Xu, J.; Huang, H.; Li, D.; Yue, Z.; Feng, C. Highly Efficient and Mild Electrochemical Mineralization of Long-Chain Perfluorocarboxylic Acids (C9–C10) by Ti/SnO_2–Sb–Ce, Ti/SnO_2–Sb/Ce–PbO_2 , and Ti/BDD Electrodes. *Environ. Sci. Technol.* **2013**, *47* (22), 13039–13046. https://doi.org/10.1021/es4034414.

(27) Liu, Y.; Fan, X.; Quan, X.; Fan, Y.; Chen, S.; Zhao, X. Enhanced Perfluorooctanoic Acid Degradation by Electrochemical Activation of Sulfate Solution on B/N

Codoped Diamond. *Environ. Sci. Technol.* **2019**, *53* (9), 5195–5201. https://doi.org/10.1021/acs.est.8b06130.

(28) Smith, S. J.; Lauria, M.; Ahrens, L.; McCleaf, P.; Hollman, P.; Bjälkefur Seroka, S.; Hamers, T.; Arp, H. P. H.; Wiberg, K. Electrochemical Oxidation for Treatment of PFAS in Contaminated Water and Fractionated Foam─A Pilot-Scale Study. *ACS EST Water* **2023**, *3* (4), 1201–1211. https://doi.org/10.1021/acsestwater.2c00660.

(29) Sukeesan, S.; Boontanon, N.; Boontanon, S. K. Improved Electrical Driving Current of Electrochemical Treatment of Per- and Polyfluoroalkyl Substances (PFAS) in Water Using Boron-Doped Diamond Anode. *Environ. Technol. Innov.* **2021**, *23*, 101655. https://doi.org/10.1016/j.eti.2021.101655.

(30) Zhu, G.; Yang, H.; Fan, X.; Quan, X.; Liu, Y. Promoting $SO_4^{\cdot-}$ and $^{\cdot}OH$ Generation from Sulfate Solution toward Efficient Electrochemical Oxidation of Organic Contaminants at a B/N-Doped Diamond Flow-Through Electrode. *Environ. Sci. Technol.* **2025**, *59* (4), 2317–2326. https://doi.org/10.1021/acs.est.4c12215.

(31) Ackerman Grunfeld, D.; Jones, A. M.; Sun, J.; Le, S. T.; Pickford, R.; Huang, Q.; Manefield, M.; Kumar, N.; Lee, M. J.; O'Carroll, D. M. Electrochemical Degradation of a C6-Perfluoroalkyl Substance (PFAS) Using a Simple Activated Carbon Cathode. *Environ. Sci. Water Res. Technol.* **2024**, *10* (1), 272–287. https://doi.org/10.1039/D3EW00543G.

(32) Liu, Y.; Chen, S.; Quan, X.; Yu, H.; Zhao, H.; Zhang, Y. Efficient Mineralization of Perfluorooctanoate by Electro-Fenton with H_2O_2 Electro-Generated on Hierarchically Porous Carbon. *Environ. Sci. Technol.* **2015**, *49* (22), 13528–13533. https://doi.org/10.1021/acs.est.5b03147.

(33) Wang, Y.; Zhao, M.; Hou, C.; Chen, W.; Li, S.; Ren, R.; Li, Z. Efficient Degradation of Perfluorooctanoic Acid by Solar Photo-Electro-Fenton like System Fabricated by MOFs/Carbon Nanofibers Composite Membrane. *Chem. Eng. J.* **2021**, *414*, 128940. https://doi.org/10.1016/j.cej.2021.128940.

(34) Zhang, X.; Wu, H.; Wang, Y.; Wang, Y.; Deng, Y.; Zhu, Y.; Zhang, S.; Rijnaarts,

H.; Bruning, H.; Qin, J.; Lin, Q.; Ni, Z.; Qiu, R. Enhancing Electrochemical Reductive Defluorination of PFASs Using ZIF-67 Modified Cathode: Mechanistic Insights and Performance Optimization. *Water Res.* **2025**, *268*, 122625. https://doi.org/10.1016/j.watres.2024.122625.

(35) Baldaguez Medina, P.; Cotty, S.; Kim, K.; Elbert, J.; Su, X. Emerging Investigator Series: Electrochemically-Mediated Remediation of GenX Using Redox-Copolymers. *Environ. Sci. Water Res. Technol.* **2021**, *7* (12), 2231–2240. https://doi.org/10.1039/D1EW00544H.

(36) Barisci, S.; Suri, R. Electrooxidation of Short and Long Chain Perfluorocarboxylic Acids Using Boron Doped Diamond Electrodes. *Chemosphere* **2020**, *243*, 125349. https://doi.org/10.1016/j.chemosphere.2019.125349.

(37) Kim, N.; Elbert, J.; Shchukina, E.; Su, X. Integrating Redox-Electrodialysis and Electrosorption for the Removal of Ultra-Short- to Long-Chain PFAS. *Nat. Commun.* **2024**, *15* (1), 8321. https://doi.org/10.1038/s41467-024-52630-w.

(38) Maldonado, V. Y.; Schwichtenberg, T.; Schmokel, C.; Witt, S. E.; Field, J. A. Electrochemical Transformations of Perfluoroalkyl Acid (PFAA) Precursors and PFAAs in Landfill Leachates. *ACS EST Water* **2022**, *2* (4), 624–634. https://doi.org/10.1021/acsestwater.1c00479.

(39) Olvera-Vargas, H.; Wang, Z.; Xu, J.; Lefebvre, O. Synergistic Degradation of GenX (Hexafluoropropylene Oxide Dimer Acid) by Pairing Graphene-Coated Ni-Foam and Boron Doped Diamond Electrodes. *Chem. Eng. J.* **2022**, *430*, 132686. https://doi.org/10.1016/j.cej.2021.132686.

(40) Pierpaoli, M.; Szopińska, M.; Wilk, B. K.; Sobaszek, M.; Łuczkiewicz, A.; Bogdanowicz, R.; Fudala-Książek, S. Electrochemical Oxidation of PFOA and PFOS in Landfill Leachates at Low and Highly Boron-Doped Diamond Electrodes. *J. Hazard. Mater.* **2021**, *403*, 123606. https://doi.org/10.1016/j.jhazmat.2020.123606.

(41) Uwayezu, J. N.; Carabante, I.; Lejon, T.; Van Hees, P.; Karlsson, P.; Hollman, P.; Kumpiene, J. Electrochemical Degradation of Per- and Poly-Fluoroalkyl

Substances Using Boron-Doped Diamond Electrodes. *J. Environ. Manage.* **2021**, *290*, 112573. https://doi.org/10.1016/j.jenvman.2021.112573.
